# First‐in‐class inhibitors of SbnA reduce siderophore production in *Staphylococcus aureus*

**DOI:** 10.1111/febs.70076

**Published:** 2025-04-02

**Authors:** Sarah Hijazi, Monica Cozzi, Somayeh Asgharpour, Omar De Bei, Serena Faggiano, Francesco Marchesani, Luca Ronda, Marialaura Marchetti, Eleonora Gianquinto, Mariacristina Failla, Gauthier Trèves, Loretta Lazzarato, Francesca Spyrakis, Barbara Campanini, Emanuela Frangipani, Stefano Bettati

**Affiliations:** ^1^ Department of Biomolecular Sciences University of Urbino Carlo Bo Italy; ^2^ Department of Medicine and Surgery University of Parma Italy; ^3^ Department of Drug Science and Technology University of Turin Italy; ^4^ Department of Food and Drug University of Parma Italy; ^5^ Institute of Biophysics National Research Council (CNR) Pisa Italy; ^6^ Biopharmanet‐Tec University of Parma Italy

**Keywords:** antimicrobial resistance, iron metabolism, SbnA, siderophores, *Staphylococcus aureus*, staphyloferrin B

## Abstract

Siderophore production, along with heme scavenging by hemophores, is one of the main mechanisms exploited by bacteria to achieve an adequate iron supply. *Staphylococcus aureus* produces two main siderophores, staphyloferrin A (SA) and staphyloferrin B (SB), with the latter produced only by the most invasive, coagulase‐positive *S. aureus* strains. Along the seven steps of the SB biosynthetic pathway, *N*‐(2‐amino‐2‐carboxyethyl)‐l‐glutamate synthase (SbnA) catalyzes the crucial formation of the intermediate *N*‐(2‐amino‐2‐carboxyethyl)‐l‐glutamate from O‐phospho‐L‐serine and glutamate. Our functional characterization of the enzyme highlighted that citrate inhibits SbnA with an inhibitory constant (*K*_i_) in the order of magnitude of the physiological concentration of the metabolite. We searched for inhibitors of SbnA within citrate analogues and identified 2‐phenylmaleic acid (2‐PhMA) as the best hit, with a *K*_i_ of 16 ± 2 μm and a mechanism of inhibition that is competitive with O‐phospho‐L‐serine for active site binding. The methyl ester of 2‐PhMA at a 2 mm concentration was effective in inhibiting siderophore biosynthesis in *S. aureus*. These results pave the way for the discovery of promising inhibitors of iron acquisition that might find application as innovative antimicrobials.

## Graphical abstract

The production of the iron‐chelating compound staphyloferrin B by the most invasive *S. aureus* strains is crucial for colonization, virulence, and survival in the host. Staphyloferrin B biosynthesis is carried out by eight enzymes, the Sbn system, that offer an appealing source of potential targets for the development of antimicrobials. We successfully identified an effective, permeable inhibitor of SbnA that decreases the production of staphyloferrin B by *S. aureus* under iron‐depleted conditions.

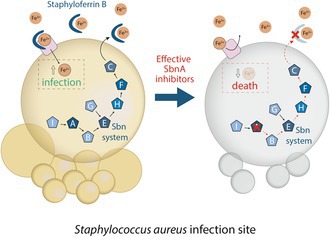

**Abbreviations**
2‐PhMA: 2‐phenylmaleic acid
2‐PhSA: 2‐phenylsuccinic acid
ACEGA: *N*‐(2‐amino‐2‐carboxyethyl)‐l‐glutamate
AMMP: 2‐amino‐6‐mercapto‐7‐methylpurine
CAS: chrome azurol S‐Fe(III)‐hexadecyltrimethylammonium bromide
DIP: 2,2′‐dipyridyl
IFD: induced fit docking
KPLP: *N*′‐pyridoxal‐lysine‐5′‐monophosphate
l‐Dap: l‐2,3‐diaminopropionic acid
l‐Glu: l‐glutamate
l‐OPS: O‐phospho‐L‐serine
MD: molecular dynamics
MESG: 7‐methyl‐6‐thioguanosine
NIS: NRPS‐independent siderophore
NRPS: nonribosomal peptide synthetase
PDB: Protein Data Bank
PLP: pyridoxal 5′‐phosphate
PNP: purine nucleoside phosphorylase
RMSD: root mean square deviation
RMSF: root mean square fluctuation
SA: staphyloferrin A
SB: staphyloferrin B
TCEP: tris(2‐carboxyethyl) phosphine hydrochloride
TMS: tris minimal succinate
TSA: tryptic soy agar
α‐AA: α‐aminoacrylate
α‐KG: α‐ketoglutarate

## Introduction

The acquisition of iron from the host is one of the main factors allowing successful colonization and infection by pathogenic bacteria. Indeed, the large number of processes involving iron, for example, Krebs cycle and oxidative phosphorylation, makes this element crucial for life. Being free iron essentially unavailable, bacteria have evolved very sophisticated systems to get this nutrient from the host's proteins in the form of either hemic or nonhemic iron. Hemic iron is mainly acquired from Hb, while nonhemic iron main sources are the proteins lactoferrin and transferrin. Molecules that allow the scavenging of iron from nonhemic sources are called siderophores, low molecular weight chelators with dissociation constants for Fe^3+^ down to 10^−52^ m [1, 2], that allow to successfully outcompete host proteins.

Staphyloferrin A (SA) and staphyloferrin B (SB) are the two main *Staphylococcus aureus* siderophores [3, 4] whose key role in iron acquisition is confirmed by the fact that they are classified as virulence factors [5] and virulent *S. aureus* strains produce as much as 100‐fold more siderophores compared to nonvirulent counterparts. While SA is produced by both coagulase‐positive and coagulase‐negative strains, SB is produced by the most invasive *S. aureus* strains [6].

**Fig. 1:**
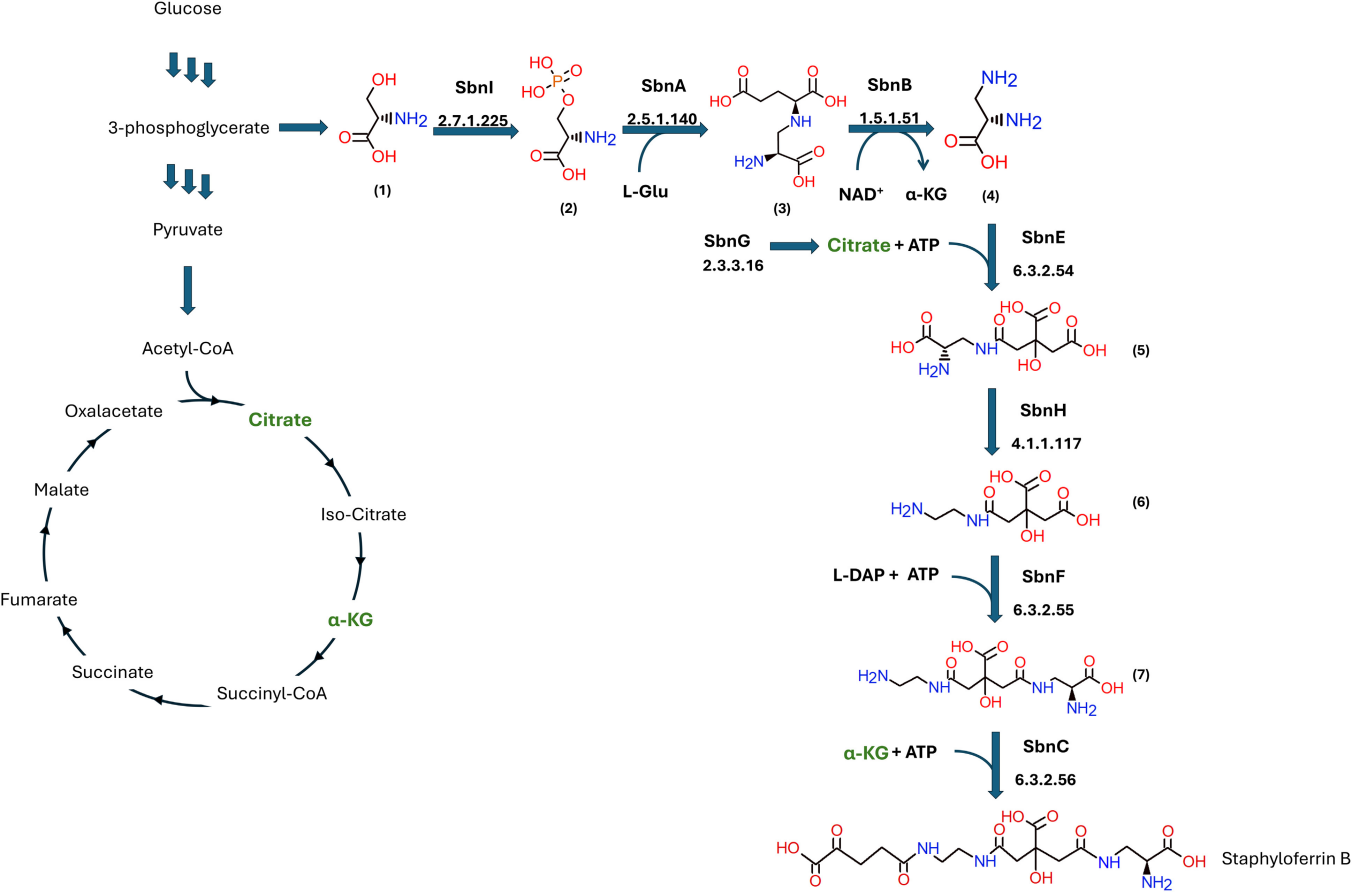
Biosynthesis of staphyloferrin B in *S. aureus*. The structures reported correspond to: (1) l‐serine, (2) O‐phospho‐L‐serine (l‐OPS), (3) *N*‐(2‐amino‐2‐carboxyethyl)‐l‐glutamate (ACEGA), (4) l‐2,3‐diaminopropionic acid (l‐Dap), (5) 2‐[(l‐alanin‐3‐ylcarbamoyl)methyl]‐2‐hydroxybutanedioate, (6) 2‐[(2‐aminoethylcarbamoyl)methyl]‐2‐hydroxybutanedioate, (7) 2‐[(l‐alanin‐3‐ylcarbamoyl)methyl]‐3‐(2‐aminoethylcarbamoyl)‐2‐hydroxypropanoate. The stereochemistry refers to the one reported in the KEGG website (www.genome.jp/pathway/sae00997). α‐ketoglutarate (α‐KG) and citrate (in green) are Krebs cycle intermediates. Citrate can also be provided independently from the Krebs cycle by the activity of SbnG.

The genes for SB biosynthesis are all encoded in the *sbn* operon (*sbnA‐I*), whose expression is under the control of the ferric uptake regulator (Fur). SB is produced from a molecule of citrate, one of α‐ketoglutarate (α‐KG), and two molecules of the nonproteinogenic amino acid l‐2,3‐diaminopropionic acid (l‐Dap) (Fig. 1).

It is interesting to notice how the pathway presents two enzymatic activities that are redundant with respect to the central metabolism, that is, SbnI, which catalyzes the phosphorylation of l‐serine to produce *O*‐phospho‐l‐serine (l‐OPS) in redundancy with the phosphorylated pathway, and SbnG that produces citrate in redundancy with the Krebs cycle. This observation suggests a critical role of the SB biosynthetic pathway under conditions that require disengagement of iron acquisition from the metabolic status.

**Fig. 2:**
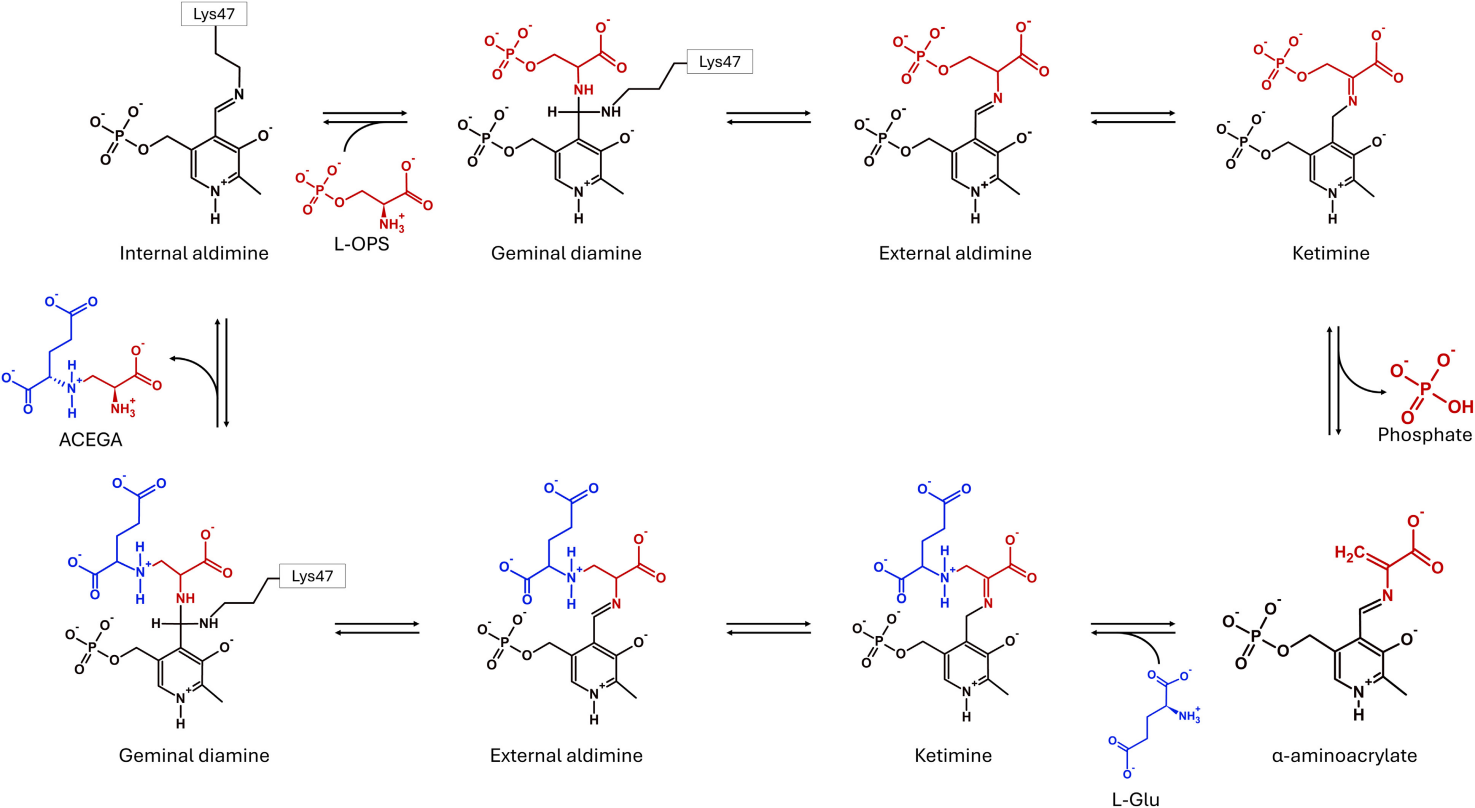
Reaction mechanism of SbnA. The first substrate, O‐phospho‐L‐serine (l‐OPS), is in red; the second substrate, l‐glutamate (l‐Glu), is in blue. The portions of ACEGA derived from l‐OPS and l‐Glu are in red and blue, respectively.

SbnA (EC number 2.5.1.140) is a pyridoxal 5′‐phosphate (PLP)‐dependent enzyme that catalyzes a β‐substitution reaction of the phosphate group of l‐OPS with l‐glutamate to produce free inorganic phosphate and N‐(2‐amin‐o‐2‐carboxyethyl‐L‐glutamate‐(ACEGA) [7, 8]. Based on the functional and structural homology with the well‐characterized PLP‐dependent enzyme *O*‐acetylserine sulfhydrylase, Kobylarz *et al*. proposed a ping‐pong mechanism for the reaction catalyzed by SbnA (Fig. 2). l‐OPS binds to the enzyme and the elimination of the phosphate group causes the formation of the α‐aminoacrylate (α‐AA) intermediate, which is then attacked by glutamate to form ACEGA.

**Fig. 3:**
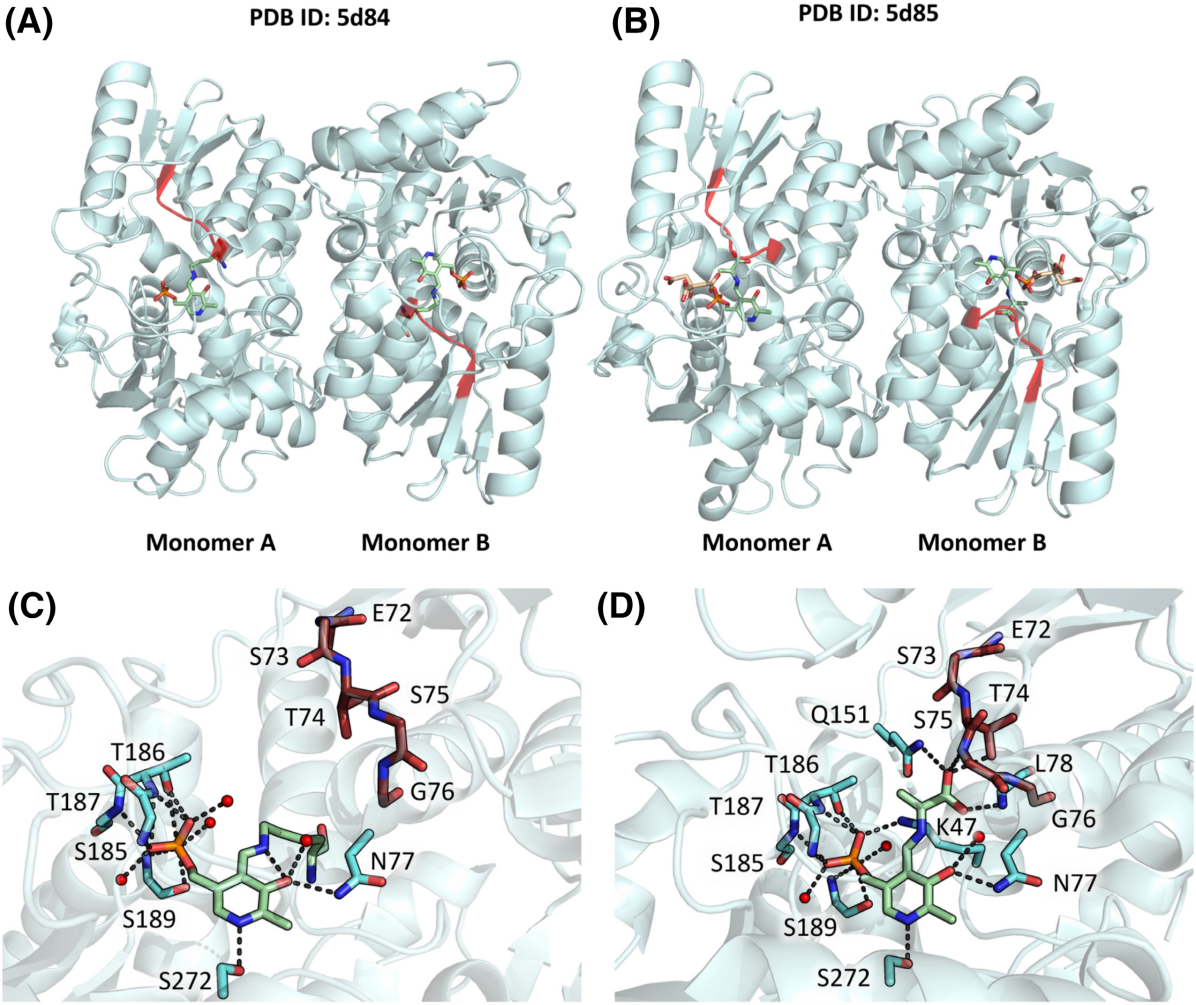
Three‐dimensional structure of SbnA in the open and closed conformation. (A) SbnA in the open conformation, PDB ID: 5D84. (B) SbnA in the closed conformation, PDB ID: 5D85. The active site loop (residues 72–78) is highlighted in red, and the pyridoxal 5′‐phosphate (PLP) is represented as green sticks; the citrate molecule is shown as beige sticks. (C) Close‐up of SbnA in the open conformation as reported in panel A with PLP (shown as green sticks) bound to Lys47 as internal aldimine. (D) Close‐up of SbnA in the closed conformation, as reported in panel B with PLP (shown as green sticks) forming the α‐aminoacrylate (α‐AA) intermediate. Active site residues are depicted as cyan sticks, active site loop residues (72 to 78) as red sticks, water molecules as red spheres, and hydrogen bonds formed by the PLP as gray dotted lines.

Four structures of the enzyme have been deposited in the PDB by Kobylarz *et al*., two corresponding to the *wt* enzyme in two catalytic intermediate states (internal aldimine and α‐AA, PDB ID 5D84 and 5D85, respectively), and two variants carrying substitutions in active site residues (Y152F and Y152F/S185G, PDB ID 5D86 and 5D87, respectively) [8]. SbnA forms a dimer showing the typical fold type II of PLP‐dependent enzymes, with a large C‐terminal domain and a small N‐terminal domain composed of a core β‐sheet surrounded by α‐helices (Fig. 3).

PLP binds in the cavity between the two domains, forming a Schiff base with Lys47 located at the base of the cleft, which is surrounded by positively charged residues. The soaking of SbnA crystals with l‐OPS led to the trapping of the α‐AA intermediate, accompanied by a rotation of the large domain towards the small domain that restricts access to the active site within a closed conformation. This rotation causes the movement of the active site loop (shown in red in Fig. 3B), comprising residues 72–78 and containing the highly conserved Ser75 directly above the α‐AA. This conformational change leads to the formation of a binding pocket between the two domains right above the α‐AA. A molecule of citrate, that was a component of the crystallization solution, was serendipitously found bound to this site but its effect on activity and dynamics was not further explored.

Although *S. aureus* might access heme‐iron in the absence of siderophores, the rationale of inhibiting SbnA lies in the well‐documented crucial role of SB for *S. aureus* colonization, virulence, and survival in preclinical models of infection [9, 10, 11]. Inhibition of siderophore biosynthesis offers an appealing, yet poorly exploited, opportunity for a better comprehension of the mechanisms that drive infectivity and for the development of antimicrobials. The only example reported to date of successful targeting of an enzyme encoded by the *sbn* operon is represented by the discovery of baulamycin A, a competitive inhibitor of SbnE that binds to the citrate‐binding site [12], presumably by mimicking its structure via the 1,3,5‐triol moiety [13]. Recent data suggest that the antibiotic effect of this compound might be mainly due to membrane disruption, although further studies are required to better elucidate this point [13].

The aim of this work is the identification of SbnA inhibitors starting from the observation that citrate can inhibit the enzyme at physiologically relevant concentrations. A more potent citrate‐mimicking compound, that is, 2‐phenylmaleate, was functionally characterized and showed a promising inhibitory activity of siderophore biosynthesis in *S. aureus*.

## Results and Discussion

### SbnA follows a ping‐pong mechanism and is inhibited by citrate, an essential intermediate in SB biosynthesis

#### Spectroscopic evaluation of ligand binding to SbnA

**Fig. 4:**
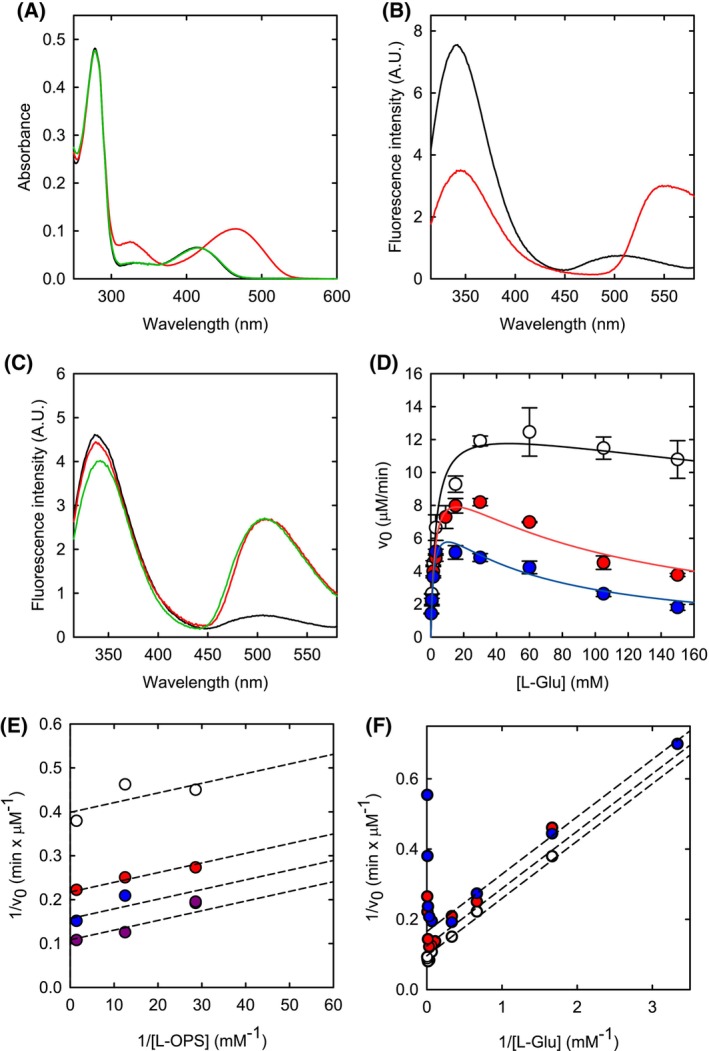
Spectroscopic properties and kinetic characterization of SbnA. (A) Absorption spectra of 14 μm SbnA in the absence of substrates (black line), and after the sequential addition of 0.035 mm O‐phospho‐L‐serine (l‐OPS) (red line) and 30 mm l‐glutamate (l‐Glu) (green line). (B) Fluorescence emission spectra of SbnA upon excitation at 298 nm in the absence (black line) and in the presence of 0.3 mm l‐OPS (red line). (C) Fluorescence emission spectra of SbnA upon excitation at 298 nm in the absence (black line) and in the presence of either 125 mm l‐Glu (red line) or 13 mm α − ketoglutarate (α − KG) (green line). (D) The kinetic parameters of the reaction catalyzed by SbnA were determined from the dependence of the initial velocity on l‐Glu concentration in the presence of 0.720 mm (open circles), 0.080 mm (red circles), and 0.035 mm (blue circles) l‐OPS. The data are the average of two independent measurements, and the error bars represent the standard deviation. Lines through data points are the fitting to Eqn (1), that allows for determining *K*_m_, *k*_cat_, and *K*_i_. (E) Double reciprocal plot of activity data. l‐Glu concentration was kept constant at 0.6 mm (open dots), 1.5 mm (red dots), 3 mm (blue dots) and 15 mm (purple dots). Lines through data points correspond to the fitting to the equation for a ping‐pong mechanism in the reciprocal form. (F) Double reciprocal plot of activity data reported in 4D. l‐OPS concentration was maintained constant at 0.035 mm (blue circles), 0.080 mm (red circles), and 0.720 mm (open circles). Lines through data points correspond to the fitting to the equation for a ping‐pong mechanism in the reciprocal form.

The reaction of SbnA with its substrates l‐OPS and l‐Glu can be monitored either by absorption or fluorescence emission spectroscopy exploiting the PLP cofactor. Indeed, the PLP absorption spectrum in the visible region depends on the catalytic intermediate and the fluorescence emission spectrum is also sensitive to the cofactor microenvironment, allowing to follow not only the reaction with the substrates but also the binding of ligands that promote conformational and/or polarity changes at the active site. The addition of l‐OPS to the enzyme causes the disappearance of the typical absorption peaks of the internal aldimine (centered at 330 and 412 nm) and the appearance of the peaks of the α‐AA intermediate at 324 and 467 nm (Fig. 4A). The peak at lower wavelengths, which is attributed to the enolimine tautomer of the α‐AA, is more intense than the peak of the enolimine tautomer of the internal aldimine present in the absence of l‐OPS, likely because of a less polar environment experienced by the cofactor in the α‐AA form.

The subsequent addition of l‐Glu to SbnA closes the catalytic cycle with the reformation of the internal aldimine. The fluorescence emission spectrum of SbnA for the excitation of the tryptophan residues at 298 nm shows two peaks (Fig. 4B,C), as already reported for other PLP‐dependent enzymes [14, 15]. The more intense peak, centered at 345 nm, is due to the direct emission of Trp residues, while the second peak, at about 500 nm, is originated by the fluorescence energy transfer between tryptophans and PLP. The addition of l‐OPS leads to a decrease in the emission intensity of the Trp residues and an increase in the emission of PLP, likely because of a more efficient energy transfer between them and the enolimine tautomer of the α‐AA (Fig. 4B). The addition of l‐Glu to the enzyme causes a threefold increase in the emission of PLP at 500 nm together with a 5 nm red shift, with no changes in the intensity of the Trp emission peak (Fig. 4C). This might be indicative of the formation of the external aldimine between l‐Glu and PLP and/or of a conformational change of the protein that leads to a perturbation of the active site polarity. The latter hypothesis seems more plausible, given that the addition of α‐KG (Fig. 4C), which is not able to form the external aldimine as it lacks the α‐amino group, leads to similar changes in the emission spectrum. The observation of direct binding of l‐Glu to the internal aldimine of the enzyme, without the formation of any covalent adduct/catalytic intermediate, supports the observation that l‐Glu gives rise to substrate inhibition (*vide infra*).

#### Catalytic parameters of SbnA and the effect of metabolic intermediates on the activity

The catalytic parameters of SbnA were calculated by global fitting of the dependence of the initial reaction velocity on both l‐Glu and l‐OPS concentrations (Fig. 4D). Since l‐Glu strongly inhibits the reaction at low l‐OPS concentrations, the dependencies were fitted to an equation accounting for substrate inhibition (Eqn 1). Substrate inhibition is frequently observed in enzymes that catalyze ping‐pong reactions and is related to the unproductive binding of the substrate to the wrong enzyme form (i.e., the internal aldimine). Interestingly, we could observe by fluorescence emission spectroscopy the binding of l‐Glu to the internal aldimine of SbnA (Fig. 4C), in support of the substrate inhibition measured here. The pattern of the double reciprocal plot (Fig. 4E,F) is compatible with different dual‐substrate mechanisms [16], with the classical ping‐pong mechanism being the most reasonable also based on the similarity of SbnA to other, well‐characterized, PLP‐dependent enzymes like tryptophan synthase and *O*‐acetylserine sulfhydrylase [17, 18]. The catalytic parameters for l‐Glu agree with those calculated by Kobylarz *et al*. [8], while the catalytic parameters for l‐OPS are significantly different, with a lower *K*_m_ and a higher *k*_cat_ that lead to a 10‐fold increase in catalytic efficiency (10^5^ m ^−1^·s^−1^; Table 1) with respect to previously published data. Considering that the pH and temperature of the assay are identical to those used in previous publications, this discrepancy might be due to the inhibitory effect of l‐Glu that went undetected in former works in which apparent catalytic parameters were calculated.

**Table 1:** Kinetic parameters of SbnA calculated by global fitting of data in Fig. 4 to Eqn (1). The *k*_cat_ value (calculated using the monomer concentration of SbnA) is 4.6 ± 0.2 s^−1^.

Substrate	*K*_m_ (mm)	*K*_i_ (mm)	*k*_cat_/*K*_m_ (M^−1^·s^−1^)
l‐OPS	0.027 ± 0.006	/	1.7 . 10^5^
l‐Glu	3.25 ± 0.36	25.75 ± 7.93	1.4 . 10^3^

**Fig. 5:**
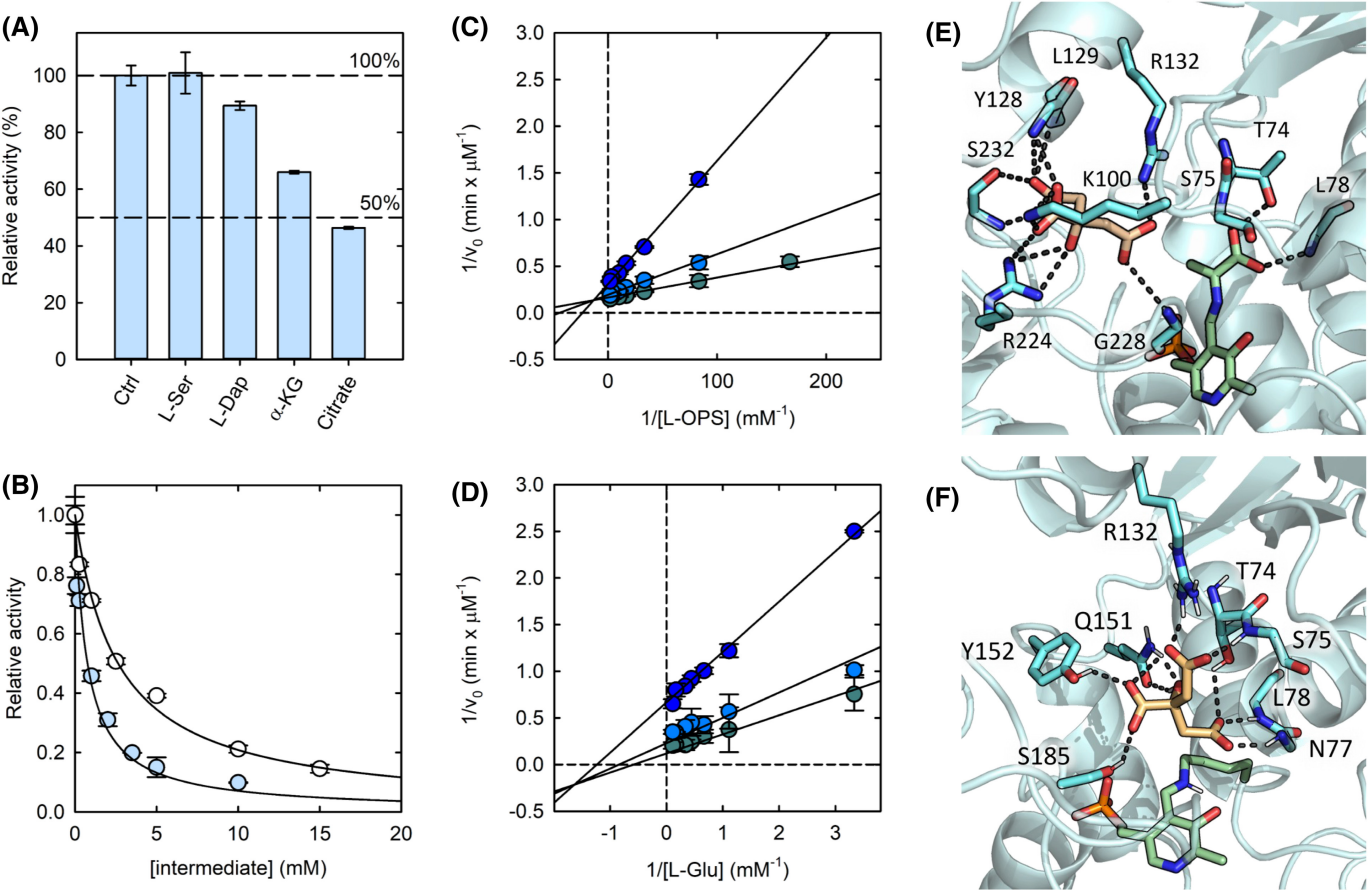
Modulation of SbnA activity by Staphyloferrin B (SB) biosynthesis intermediates. (A) Initial velocity of SbnA in the absence and presence of SB biosynthesis intermediates at 1 mm final concentration, using 0.03 mm O‐phospho‐L‐serine (l‐OPS) and 3 mm l‐glutamate (l‐Glu). The bars represent the average of two replicates. Error bars represent the standard deviation. (B) Dependence of the relative activity of SbnA on the concentration of either citrate (cyan dots) or α‐ketoglutarate (α‐KG) (open dots). l‐OPS and l‐Glu were used at concentrations equal to their *K*_m_s, that is, 0.03 and 3 mm, respectively. The lines through data points represent the fitting to Eqn (2) with IC_50_ = 0.8 ± 0.1 mm for citrate and IC_50_ = 2.6 ± 0.2 mm for α‐KG. The data represent the average of two replicates. Error bars represent the standard deviation. (C) Double reciprocal plots of the initial velocity of SbnA in the presence of 3 mm l‐Glu as a function of l‐OPS concentration in the absence of citrate (teal circles), and in the presence of either 1 mm (light blue circles) or 5 mm (blue circles) citrate. Straight lines represent the global fitting of data points to the reciprocal version of Eqn (3). The data represent the average of two replicates. Error bars represent the standard deviation. (D) Double reciprocal plots of the initial velocity of SbnA in the presence of 0.03 mm l‐OPS as a function of l‐Glu concentrations in the absence of citrate (teal circles), and in the presence of 1 mm (light blue circles) or 5 mm (blue circles) citrate. Straight lines represent the global fitting of data points to the reciprocal version of Eqn (4). Data points are the means of two replicates; error bars represent the standard deviation. (E) Crystal structure of SbnA in the closed conformation in the presence of citrate (orange sticks) (PDB ID: 5D85). (F) Docking pose of citrate (orange sticks) in the open structure of SbnA (PDB ID: 5D84): protein residues are shown as cyan sticks, pyridoxal 5′‐phosphate (PLP) bound to Lys47 as green sticks, hydrogen bonds as gray dashed lines.

It is well assessed that citrate, a key intermediate in SB biosynthesis, inhibits SbnH and SbnC, two enzymes of the pathway that operate downstream with respect to SbnA (Fig. 1). The IC_50_ for citrate inhibition of SbnH, a PLP‐dependent decarboxylase, is about 0.3 mm [19], while SbnC, a nonribosomal peptide synthetase‐independent siderophore synthetase, is inhibited by citrate even more effectively, with an IC_50_ of 0.083 mm [20]. Interestingly, the citrate concentration measured in *E. coli* and *S. aureus* grown under nutrient‐rich conditions is about 1–2 mm [21, 22], thus the biosynthesis of SB should be strongly impaired under these conditions. It is likely, however, that the iron‐sparing response of *S. aureus* deeply affects citrate concentrations to a level at which SbnH and SbnC are active again. In the case of SbnA and SbnB, both involved in the biosynthesis of key SB building blocks, inhibition by citrate, or any other intermediate of the pathway, has never been investigated, although the binding of citrate has been observed in both crystal structures [7, 8]. The only reported inhibitor for SbnA is cysteine, with a *K*_i_ of 0.090 mm, but its effect is probably related to the formation of a thiazolidine adduct with PLP [8, 23], very common in PLP‐dependent enzymes, that is, the inhibition is aspecific. Since the role of citrate in the regulation of SB biosynthesis is likely connected with changes in central metabolism during adaptation to the host environment, we deemed it interesting to test its effect, as well as that of other intermediates, on SbnA.

l‐serine and l‐Dap, two on‐pathway precursors of SB, do not substantially affect the activity of the enzyme (Fig. 5A), while citrate and α‐KG inhibit the enzyme, the former being more potent, with an IC_50_ of about 0.8 mm (Fig. 5B).

Both molecules are off‐pathway precursors of SB, and both are intermediates of the Krebs cycle. From this preliminary observation, one might conclude that a regulatory network could connect the central metabolism with SB biosynthesis. The IC_50_ of α‐KG (2.6 ± 0.2 mm, Fig. 5B) is approximately sixfold larger than the physiological concentration of this molecule in *E*. *coli* [24], thus indicating that it is not likely to exert a physiological role in the modulation of the enzyme. On the other hand, the pervasive effect of citrate on the SB biosynthesis pathway draws attention for the potential identification of inhibitors. For this reason, the mechanism of action of citrate was further investigated.

The double reciprocal plot of the dependency of the initial velocity on the concentrations of the substrates in the absence and presence of different concentrations of citrate reveals that citrate decreases apparent *V*_max_ values for both l‐Glu (at 0.03 mm l‐OPS) and l‐OPS (at 3 mm l‐glutamate) (Fig. 5C,D), increases the apparent *K*_m_ values for l‐OPS, and decreases the apparent *K*_m_ values for l‐Glu. The pattern revealed in these plots would appear consistent with an inhibition mechanism in which both the free enzyme and the α‐AA intermediate can bind citrate. The K_i_s of citrate for the two forms of the enzyme, estimated by fitting the direct data to Eqn (3), are comparable with *K*_i_ for the free enzyme of 0.9 ± 0.2 mm and *K*_i_ for the α‐AA intermediate of 2.3 ± 0.3 mm. Since the only available structure in complex with SbnA is the one carrying the α‐AA intermediate in the active site (PDB ID: 5D85, Fig. 5E), citrate was docked in the free enzyme form (PDB ID: 5D84) to find a plausible binding pose (Fig. 5F). In the top‐ranked poses, citrate is more deeply inserted inside the active site with respect to the binding position observed in the crystal structure of its complex with the α‐AA intermediate of SbnA. In the docked structure, citrate interacts with Arg132, Tyr152, and Ser185, which are the key residues identified by Kobylarz *et al*. [8] as responsible for the selective binding of l‐OPS. Moreover, citrate engages in multiple hydrogen bonds with the active site loop (residues 72–78), possibly mimicking with its carboxylic groups the interactions established by the α‐AA intermediate with the enzyme as found in the crystal structure of the closed form (Gln151; Thr74; Ser75; Leu78. PDB ID: 5D85) (Fig. 5E).

### Identification and characterization of SbnA inhibitors derived from citrate

**Fig. 6:**
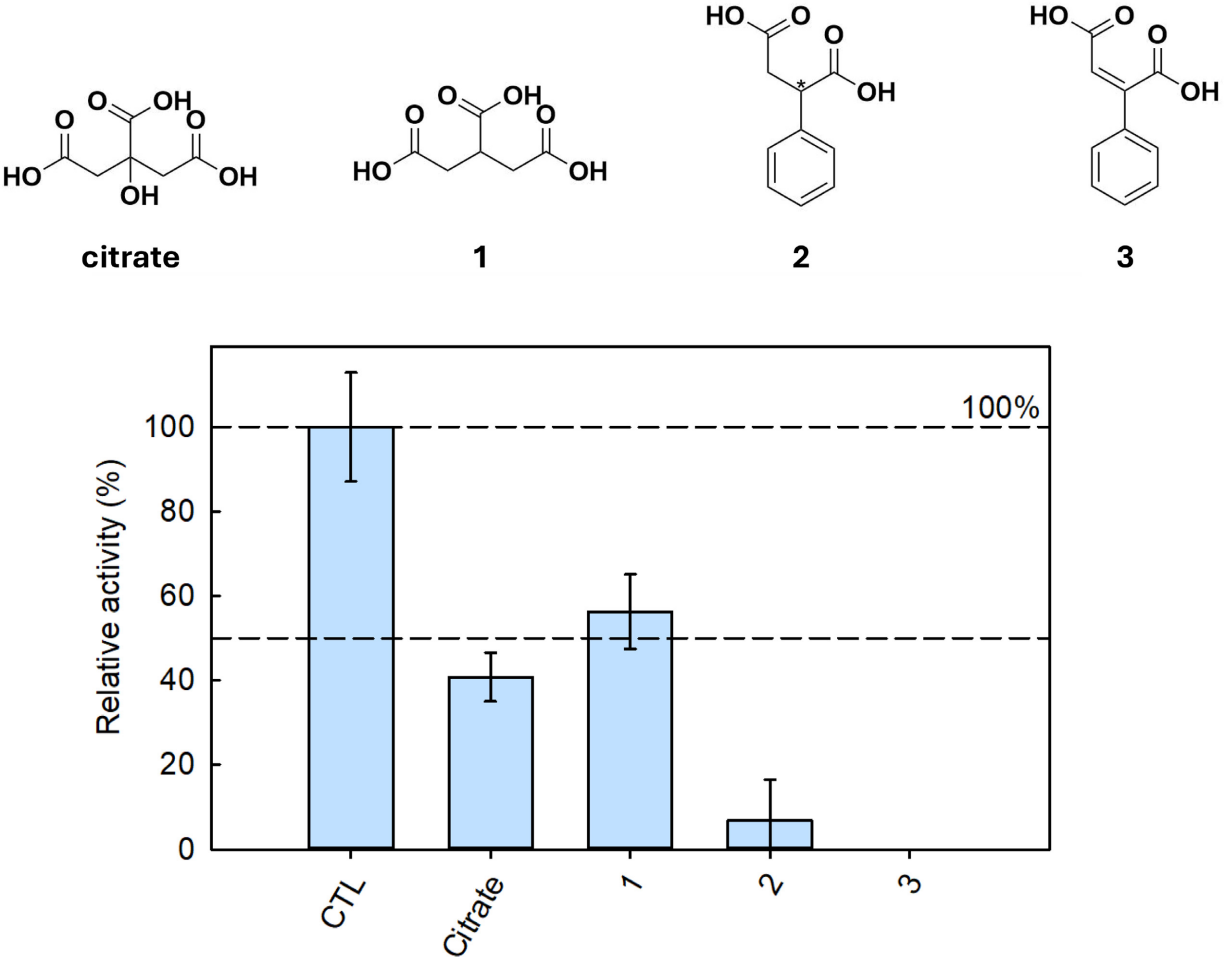
Citrate analogs structures and evaluation as possible SbnA inhibitors. (1) tricarballylic acid; (2) (R/S)‐2‐phenylsuccinic acid ((R/S‐)‐2‐PhSA); (3) 2‐phenylmaleic acid (2‐PhMA). Relative activity of SbnA alone (Ctrl), in the presence of citrate and citrate analogs **1**, **2**, and **3** at 5 mm. The bars represent the average of two duplicates, and the error bars represent the standard deviation. In the presence of compound **3**, the activity of the enzyme is completely abolished.

Considering the inhibitory activity of citrate, we wondered if citrate derivatives could behave as promising SbnA inhibitors. Thus, we tested two commercial citrate derivatives, that is, tricarballylic acid (**1**) and (R/S)‐2‐PhSA (**2**) (Fig. 6). These compounds bear at least two carboxylic groups, thus potentially interact with the active site loop and with the key residues Arg132 and Tyr152.

**Fig. 7:**
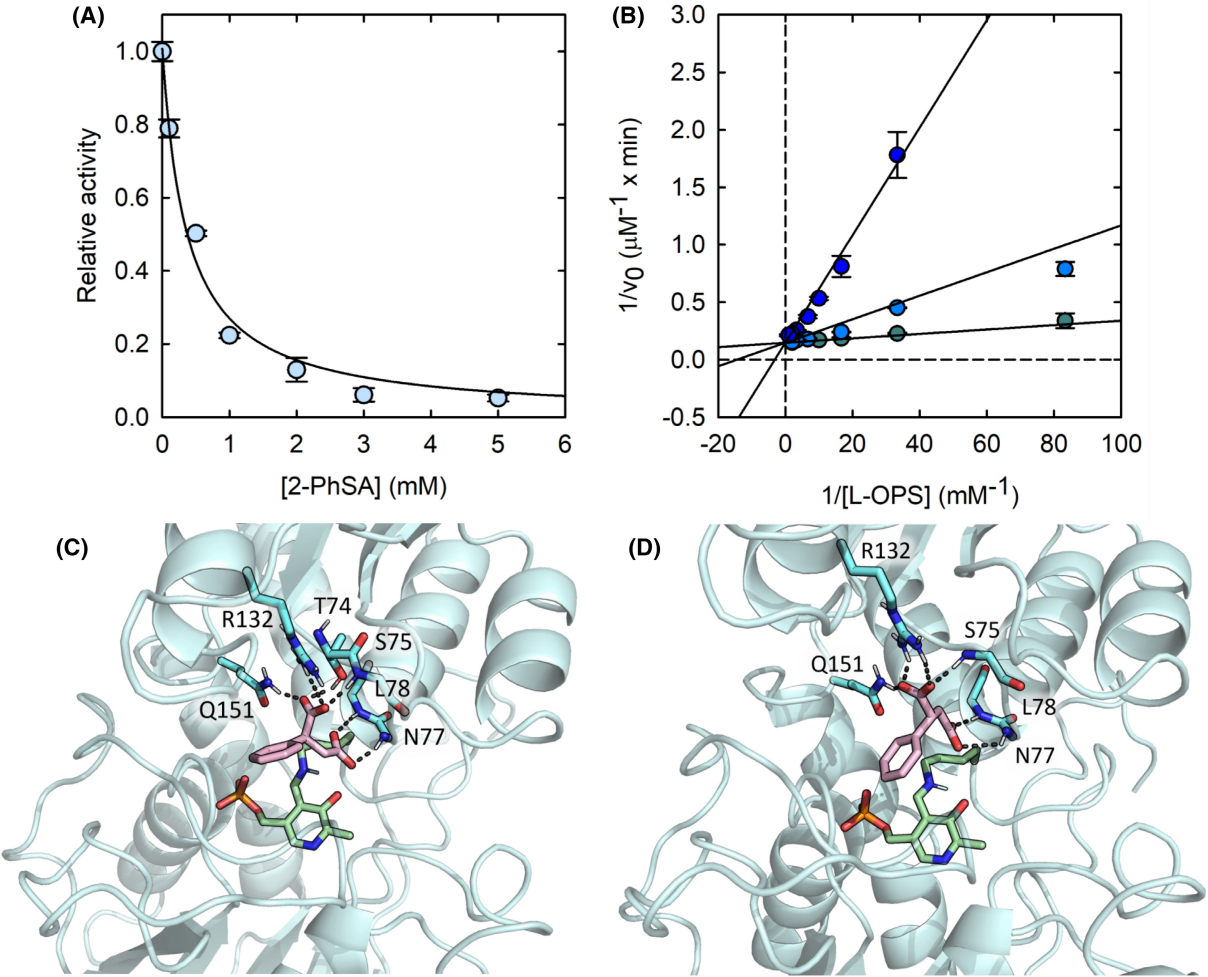
Effect of 2‐phenylsuccinic acid (2‐PhSA) on SbnA activity and docking of the molecule into the enzyme active site. (A) Dependence of the initial velocity of 50 nm SbnA as a function of 2‐PhSA concentration in the presence of 0.03 mm O‐phospho‐L‐serine (l‐OPS) and 3 mm l‐glutamate (l‐Glu). The data represent the average of two replicates. Error bars represent the standard deviation. (B) Double reciprocal plots of the initial velocity of SbnA (50 nm) in the presence of 3 mm l‐Glu as a function of l‐OPS concentrations in the absence of 2‐PhSA (teal circles), and in the presence of either 0.37 mm (light blue circles) or 2 mm 2‐PhSA (blue circles). Straight lines represent the global fitting of data points to the reciprocal form of Eqn (5). The data represent the average of two replicates; error bars represent the standard deviation. (C) Docking pose of (R)‐2‐PhSA (purple sticks) in the open structure of SbnA (PDB ID: 5D84): residues are shown as cyan sticks, *N*′‐pyridoxal‐lysine‐5′‐monophosphate (KPLP) as green sticks, hydrogen bonds as gray dashed lines. (D) Docking pose of (S)‐2‐PhSA (purple sticks) in the open structure of SbnA (PDB ID: 5D84): residues are shown as cyan sticks, KPLP as green sticks, hydrogen bonds as gray dashed lines.

The two citrate analogs were assayed at 5 mm and their activity compared with the inhibitory activity of an equimolar citrate concentration (Fig. 6). The absence of the hydroxyl group of citrate in tricarballylic acid (**1**) slightly decreases the inhibitory activity. The removal of a carboxylic moiety and the incorporation of a phenyl ring (compound **2**) led to a significant increase in the inhibitory activity with an IC_50_ of 366 ± 37 μm (Fig. 7A).

We thus investigated the mechanism of 2‐PhSA by measuring the initial velocity as a function of substrate concentration, both in the absence and presence of different inhibitor concentrations. The double reciprocal plot showed that the lines intersect the *y*‐axis at the same value of 1/*v*_0_, thus indicating that, differently from citrate, 2‐PhSA binds to the free enzyme form only, competing with the substrate l‐OPS for active site binding. The *K*_i_ calculated by fitting the direct data to Eqn (5) is 124 ± 13 μm (Fig. 7B).

**Fig. 8:**
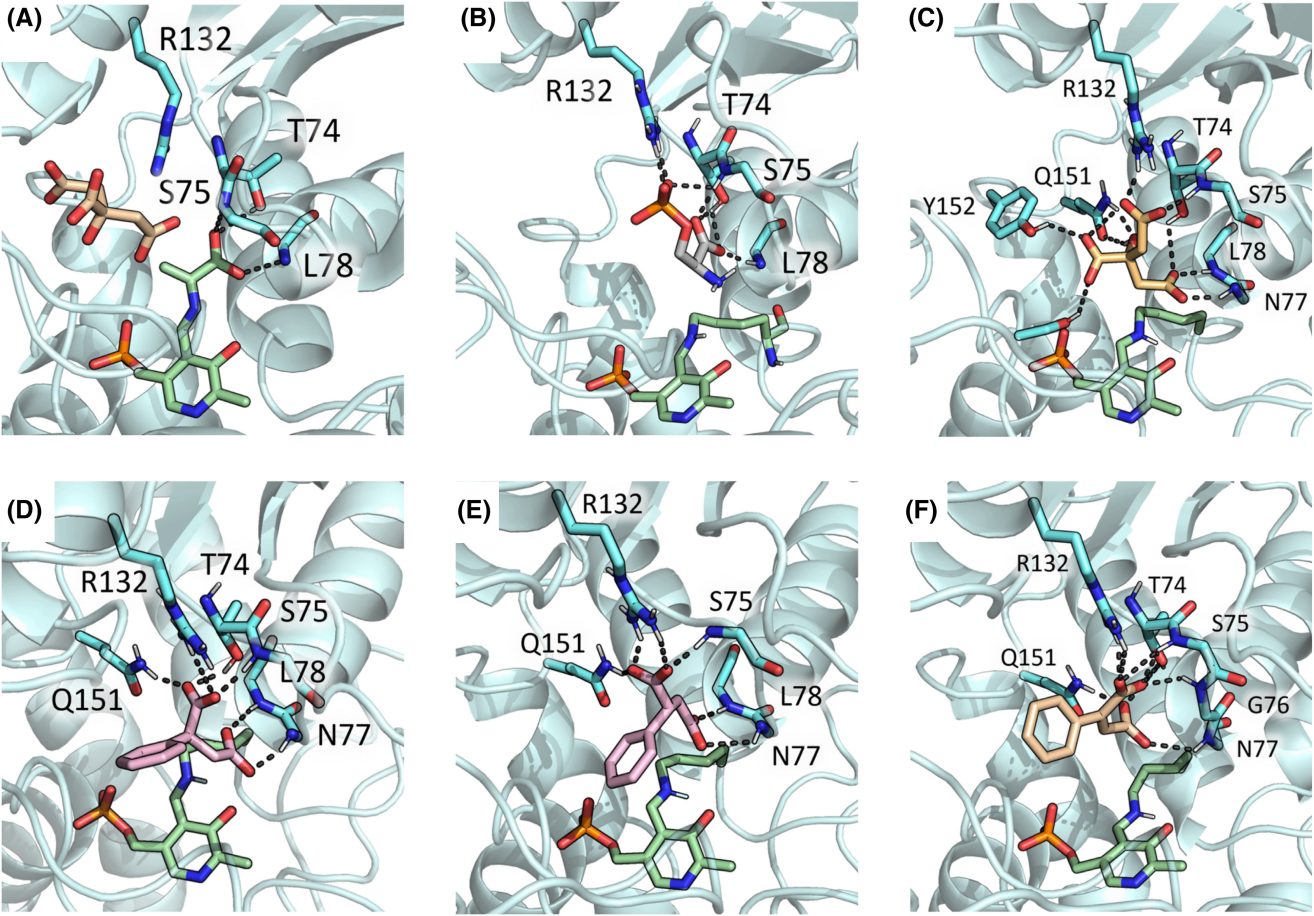
Rationalizing a common binding pose for studied SbnA ligands. The figure compares the (A) α‐aminoacrylate intermediate (light green) in PDB ID: 5D85 with docking poses of ligands in the open form of SbnA (PDB ID: 5D84): (B) docking pose of O‐phospho‐L‐serine (l‐OPS); (C) docking pose of citrate in the l‐OPS site; (D) docking pose of (R)‐compound **2**; (E) docking pose of (S)‐compound **2**; (F) docking pose of compound **3**.

To rationalize the activity of compound **2**, the docking poses of both enantiomers (R)‐2 and (S)‐2 were compared with the docking pose of citrate and with the X‐ray pose of the α‐AA intermediate (Fig. 8).

The presence of the phenyl moiety in compound **2** may increase the activity when compared to citrate, likely because it helps to orient the carboxyl groups towards the active site loop, while the many degrees of freedom of the citrate may explain its lower affinity. Both enantiomers of compound **2**, (R)‐2 and (S)‐2 showed interactions with Arg132, Gln151, and with the active site loop (Fig. 7C,D).

**Fig. 9:**
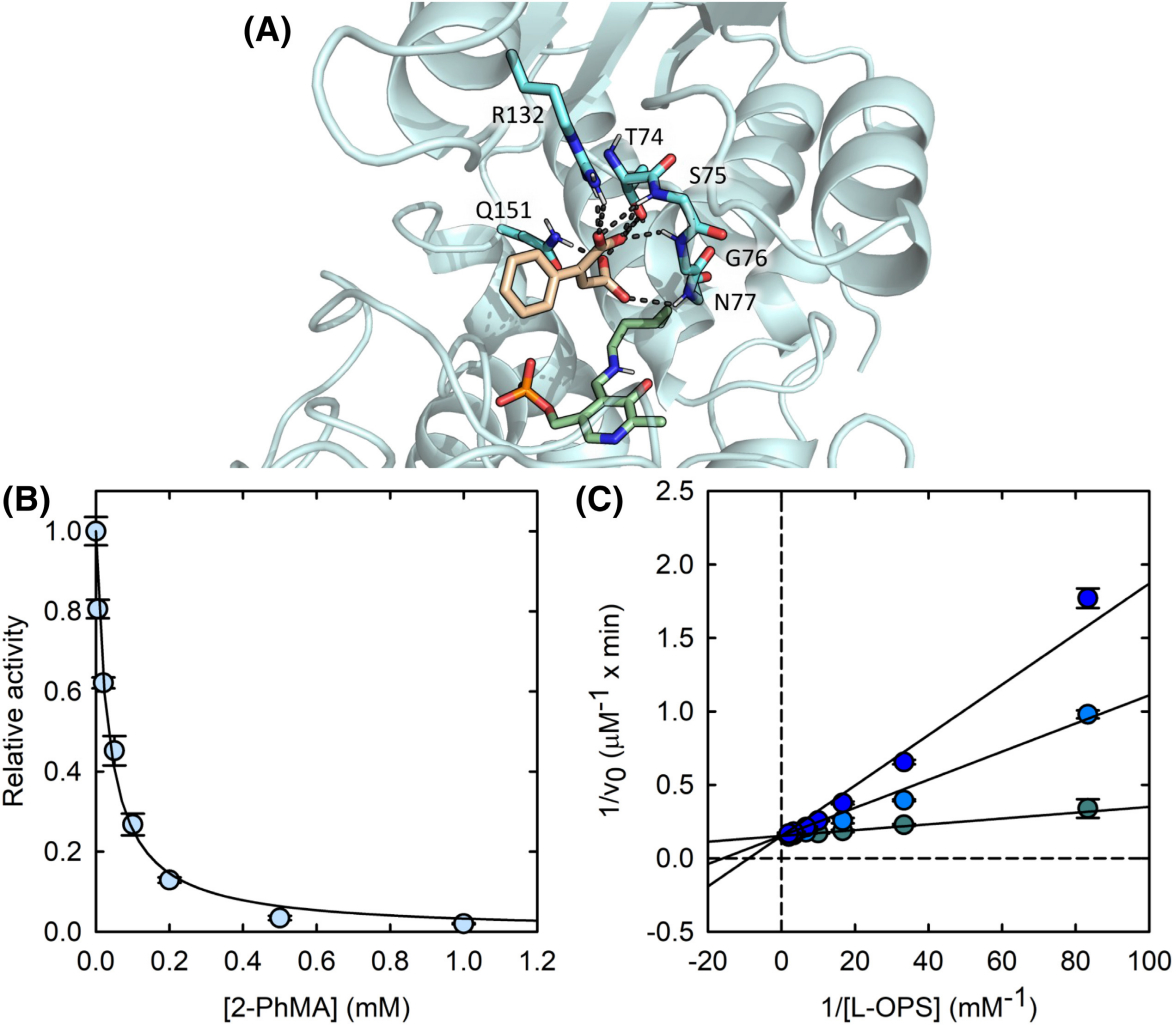
Effect of 2‐phenylmaleic acid (2‐PhMA) on SbnA activity and docking of the molecule into the enzyme active site. (A) Docking pose of 2‐PhMA (wheat sticks) in the open structure of SbnA (PDB ID: 5D84): residues are shown as cyan sticks, *N*′‐pyridoxal‐lysine‐5′‐monophosphate (KPLP) as green sticks, hydrogen bonds as gray dashed lines. (B) Dependence of the initial velocity of 50 nm SbnA as a function of 2‐PhMA concentrations in the presence of 0.03 mm O‐phospho‐L‐serine (l‐OPS) and 3 mm l‐glutamate (l‐Glu). The data represent the average of two replicates. Error bars represent the standard deviation. (C) double reciprocal plot of the initial velocity of SbnA (50 nm) in the presence of 3 mm l‐Glu as a function of l‐OPS concentrations in the absence of 2‐PhMA (teal circles), and in the presence of 0.04 mm (light blue circles) or 0.08 mm 2‐PhMA (blue circles). Straight lines represent the global fittings of data points to the reciprocal form of Eqn (5). The data represent the average of two replicates; error bars represent the standard deviation.

However, when compared with the X‐ray pose of α‐AA and the docking pose of citrate, we noticed that both compounds tend to orient one carboxyl group at the base of the active site loop, while another carboxyl group was oriented to interact with Arg132. This geometry was better respected by the (R) enantiomer of compound **2**, and we speculated that it might be key to inhibit SbnA. As such, we envisaged that the insertion of a double bond constraining the two carboxyl groups in Z‐configuration might be beneficial for activity, potentially blocking the ligand in its bioactive conformation. The resulting compound, 2‐PhMA (**3**) completely abolished the activity of the enzyme at a 5 mm concentration (Fig. 6) and was docked in SbnA, to evaluate if the rigidification introduced by the double bond could affect the docking pose. Compound **3**'s docking pose showed that the presence of the double bond in Z‐configuration may likely not affect the electrostatic and polar interactions with key residues of the active site, but greatly reduced the conformational variability observed in the docking poses of compound **2**. Indeed, compound **3** was found to be 10‐fold more potent with respect to compound **2** (IC_50_ = 37 ± 3 μm) while retaining the same mechanism of inhibition (i.e., it binds the free enzyme only, competing with l‐OPS). The *K*_i_ decreases accordingly by about 10‐fold (16 ± 2 μm; Fig. 9).

### MD simulations

To further investigate the atomic details of SbnA inhibition, we set up 1‐μs long replicas of unbiased molecular dynamics (MD) simulations of the free form of SbnA and the selected docking pose of 2‐PhMA in SbnA (2‐PhMA + SbnA). Considering that, based on the results of kinetic experiments, 2‐PhMA was only able to interact with the free enzyme, or rather the open conformation of SbnA, for all simulations the latter form of the enzyme (PDB ID: 5D84) was used as the starting structure. These simulations were intended to monitor the influence that 2‐PhMA may have on the conformation of SbnA and to address the stability of the complex system, that is, of the interactions established by 2‐PhMA in the enzyme active site.

#### Global conformational stability

**Fig. 10:**
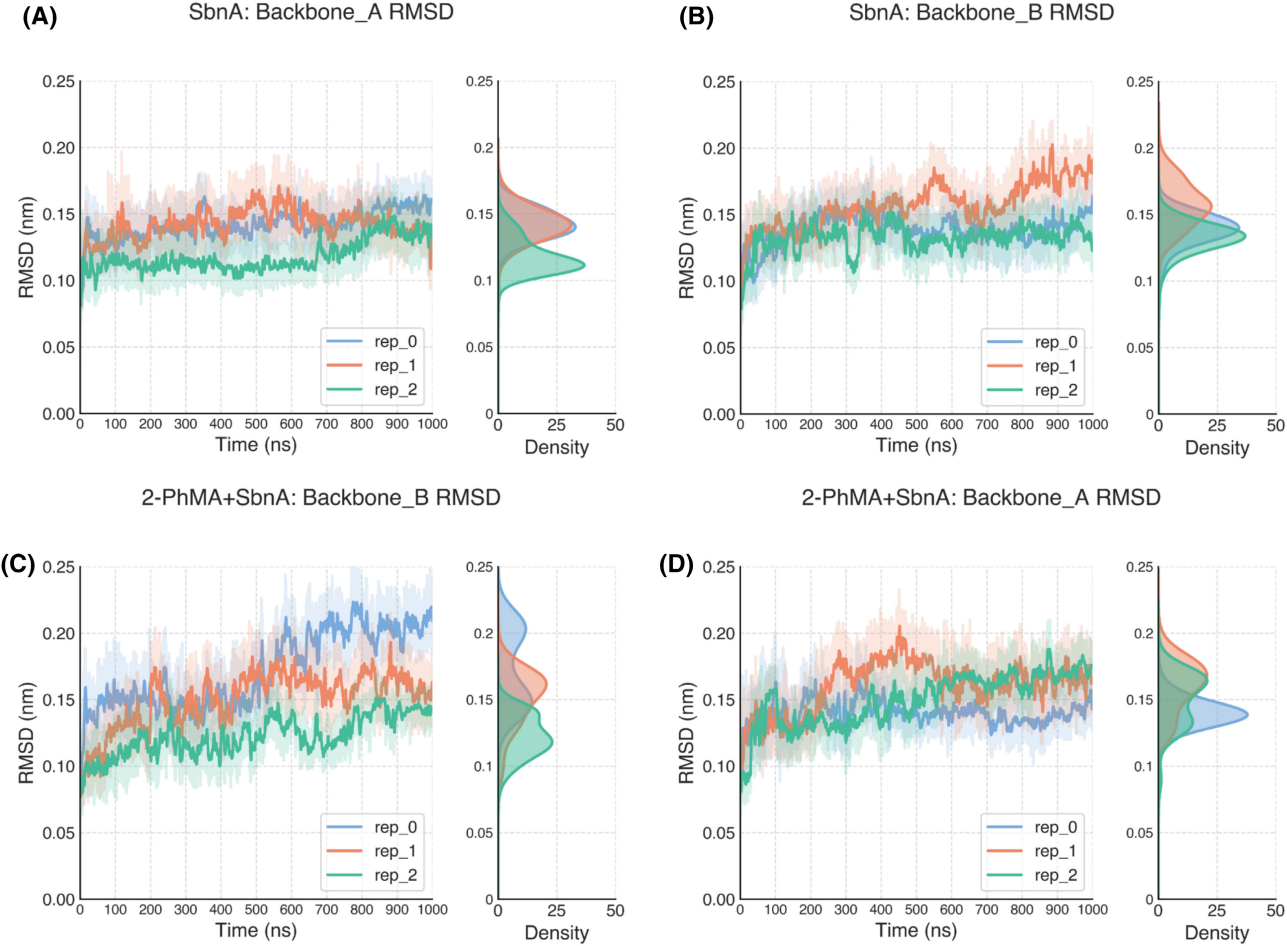
SbnA conformation in MD simulations. Root mean square deviation (RMSD) vs. simulation time of the protein backbone in SbnA (A, B) and 2‐PhMA+SbnA (C, D) systems. Three 1 μs‐long MD replicas were carried out for each system. For each frame, the RMSD with respect to the initial SbnA backbone conformation is plotted, with a rolling average (average RMSD every 500 frames) reported to improve readability. The RMSD distributions are reported on the right of each panel and were calculated for all 1‐μs long trajectories. After an initial equilibration phase, the conformation of SbnA bound to 2‐phenylmaleic acid (2‐PhMA) remained fairly stable, with an average RMSD across all replicas of 0.145 ± 0.020 nm for chain A and 0.148 ± 0.031 nm for chain B. RMSD values were slightly higher when compared to the average RMSD across all replicas of the free form of SbnA (average RMSD chain A: 0.134 ± 0.017, chain B: 0.142 ± 0.018). In only one case (2‐PhMA+SbnA, rep_0, chain B), a significant change in RMSD was observed, resulting in a higher RMSD profile. This drift in the RMSD profile was due to a conformational change of the loop included between residues 213 and 221, which was not observed in other replicas and was likely not induced by 2‐PhMA. One replica of SbnA molecular dynamics (Movie S1) and of 2‐phenylmaleic acid (2‐PhMA) + SbnA (Movie S2).

**Fig. 11:**
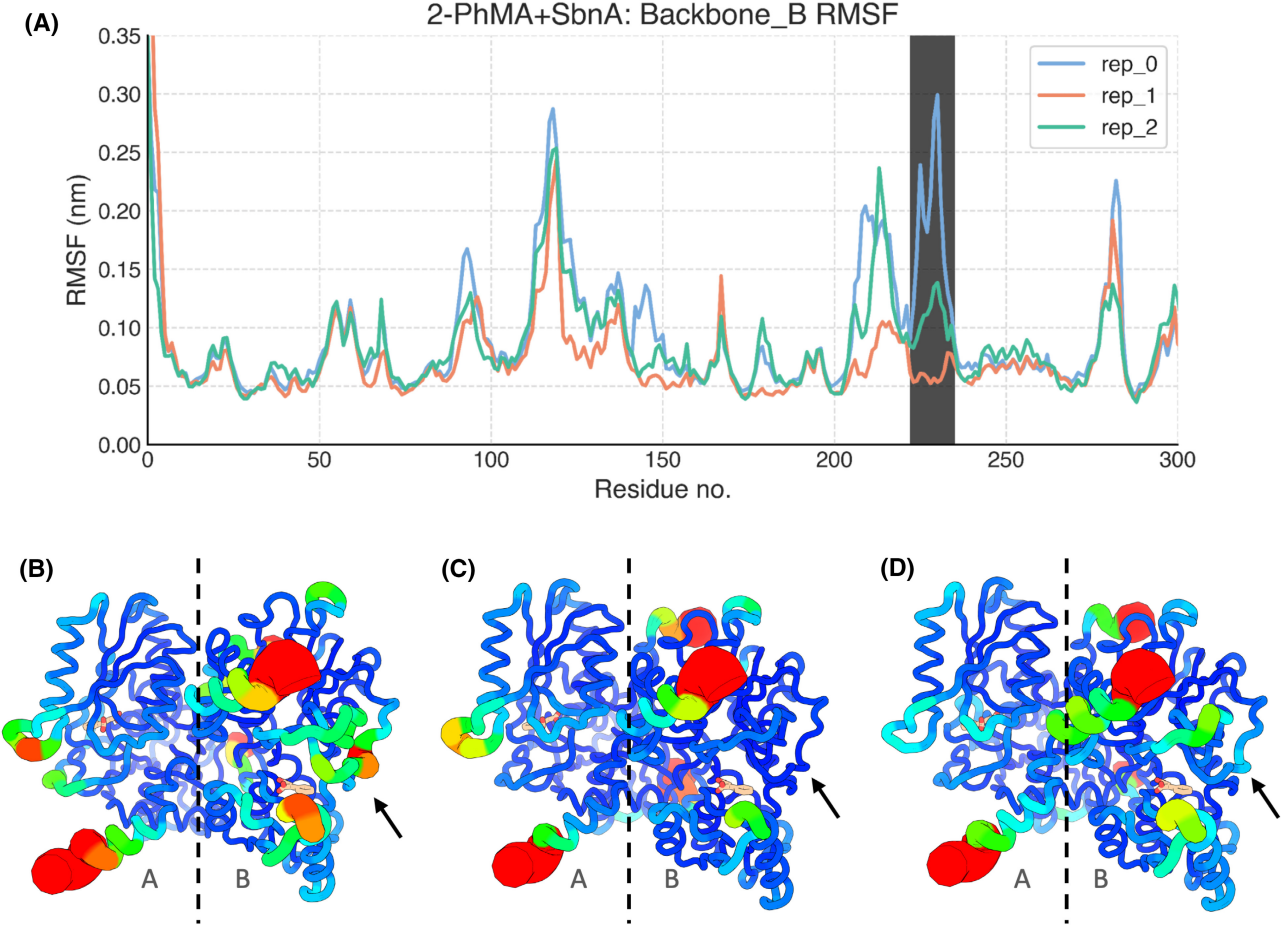
Root mean square fluctuation (RMSF) of protein backbone in chain B. The regions of SbnA associated with the conformational change highlighted by the RMSD profile in replica 0, chain B (Fig. 10) were investigated. For all replicas (three 1 μs‐long MD replicas were carried out for each system), the RMSF was calculated on the backbone atoms of chain B and plotted in panel (A). In panels (B–D), the same RMSF values are depicted as B‐factors putty representation of the SbnA dimer, for (B) replica 0, (C) replica 1, and (D) replica 2; A and B chains are labeled and separated by a dashed line, and 2‐phenylmaleic acid (2‐PhMA) is shown as sticks. In panels (B–D), colors range from blue (low RMSF) to red (high RMSF). The peak highlighted by the black shading in panel (A) indicates a loop between residues 222–235 (black arrow, panel (B–D)), which appeared particularly flexible in replica 0. However, such behavior was not observed in other replicas, as shown in panels (C, D), nor was it observed in chain A.

The root mean square deviation (RMSD) was computed on protein backbone atoms as a metric to assess conformational convergence (Figs. 10 and 11).

Having noted that during the MD simulations a conformation intermediate between the X‐ray open (PDB ID: 5D84) and closed (PDB ID: 5D85) states was stabilized, we investigated if this intermediate conformation was induced by the presence of the 2‐PhMA inhibitor. However, this intermediate conformation was also recorded in all the replicas performed in the absence of 2‐PhMA, which started from the open form of the enzyme but at the end of the simulation resembled more the closed conformation bound to the α‐AA intermediate (see Movie S1 for a direct comparison between the trajectory and the closed structure of PDB ID: 5D85). We concluded that this intermediate conformation stabilized in MD simulations was recorded regardless of the presence of the 2‐PhMA inhibitor; hence, compared to the free form of the enzyme, the overall conformation of SbnA bound to 2‐PhMA was not significantly affected by the inhibitor during the simulations.

#### Local effect of 2‐PhMA on the SbnA active site

**Fig. 12:**
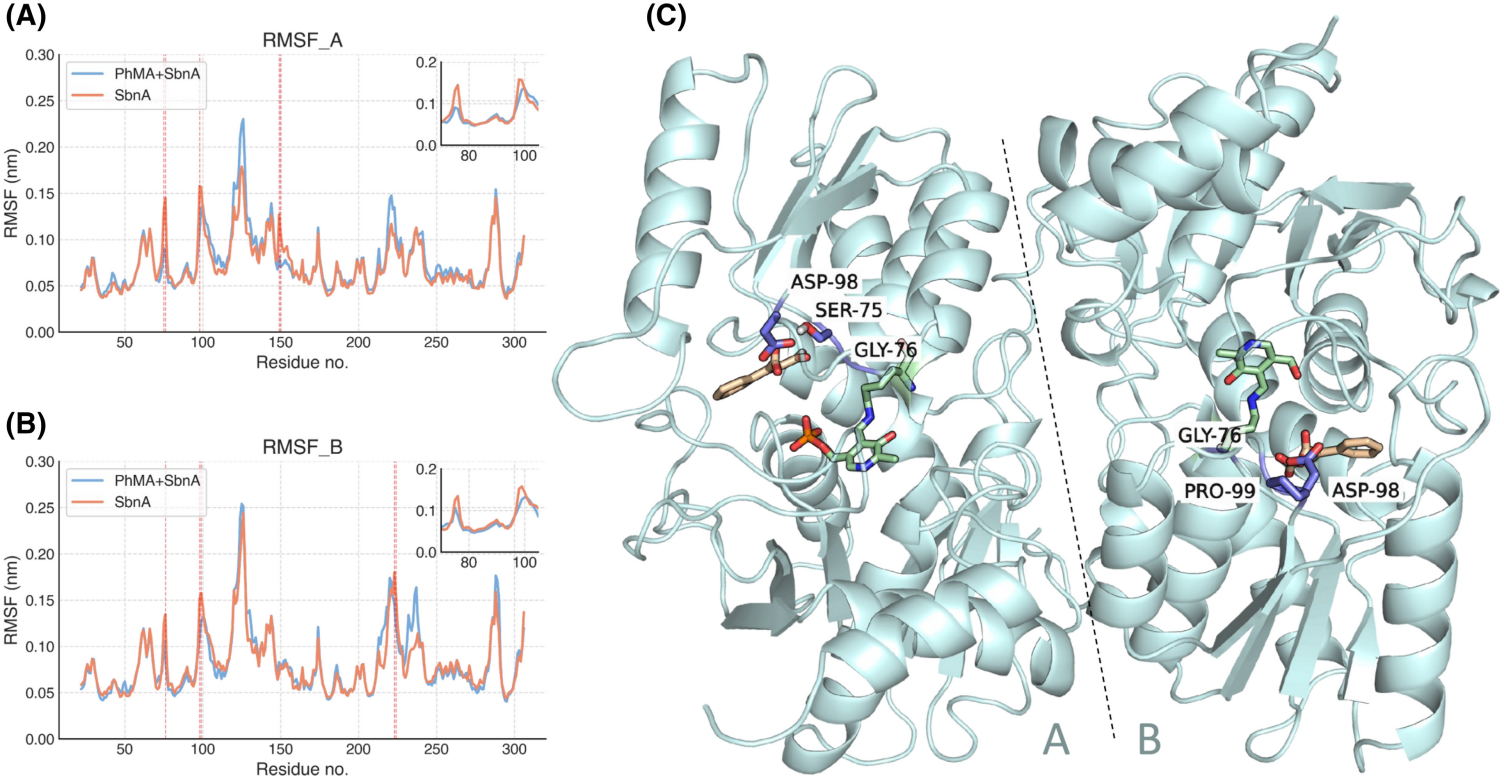
RMSF calculated on chain A and B, averaged across all replicas (three replicas, 1 μs‐long MD) of simulated systems. (A) Root mean square fluctuation (RMSF) of chain A and (B) of chain B. Residues with significantly higher RMSF in SbnA simulations are indicated with a dashed red line on the plot; the flexible N‐ and C‐termini (residues 7–15 and 300–324, respectively) have not been reported. The RMSF between the two simulated systems was considered significantly different when the average RMSF across all three replicas deviated by at least twice the standard deviation. The inset highlights the peaks corresponding to residues that were stabilized by 2‐phenylmaleic acid (2‐PhMA), namely Ser75, Gly76, and Asp98 in chain A, and residues Gly76, Asp98, and Pro99 in chain B. (C) Residues with significant RMSF differences are shown as sticks and labeled on the SbnA structure. 2‐PhMA and pyridoxal 5′‐phosphate (PLP) are reported as sticks for reference.

While RMSD provides a metric for evaluating SbnA global conformation, we focused our interest on detecting if specific residues in the SbnA binding site were stabilized by the 2‐PhMA ligand, hence reflecting persistent interactions with the inhibitor. To this aim, we further inspected the root mean square fluctuation (RMSF) averaged across all replicas for each chain (Fig. 12A,B) and identified residues with a significant difference in flexibility. In particular, we identified residues for which the RMSF was significantly higher in SbnA than in 2‐PhMA+SbnA, hence indicating ligand‐mediated stabilization effects by 2‐PhMA. Residues belonging to the l‐OPS binding site loop (Ser75‐Gly76) were stabilized by the presence of the inhibitor, as well as residues Asp98 and Pro99, belonging to another loop surrounding the l‐OPS pocket (Fig. 12C).

**Fig. 13:**
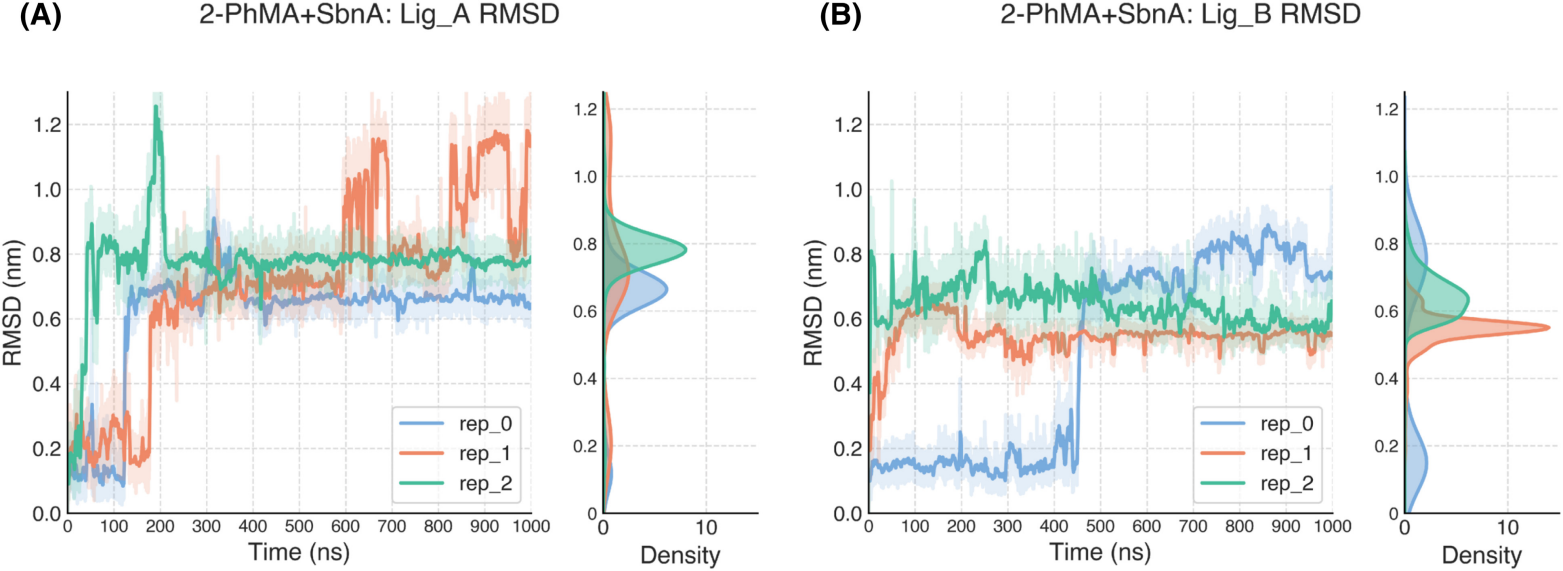
Ligand RMSD vs simulation time. We further inspected the root mean square deviation (RMSD) of the ligand in the 2‐phenylmaleic acid (2‐PhMA) + SbnA system, to track the pose of 2‐PhMA across replicas in chain A (panel A) and in chain B (panel B). For each frame, the RMSD with respect to the initial ligand conformation is plotted, with a rolling average (average RMSD every 500 frames) reported to improve readability. In all simulations (three replicas per system, 1 μs‐long molecular dynamics (MD)), after a short period of time, the pose deviated from the docked one and reached another pose that remained stable for the rest of the simulation (see Movie S2), except for one case in which 2‐PhMA significantly moved away from the O‐phospho‐L‐serine (OPS) site (rep_1, chain A in panel A). However, even with a different pose, 2‐PhMA remained in the OPS binding site in all but two cases (replica 1–2 chain A).

**Fig. 14:**
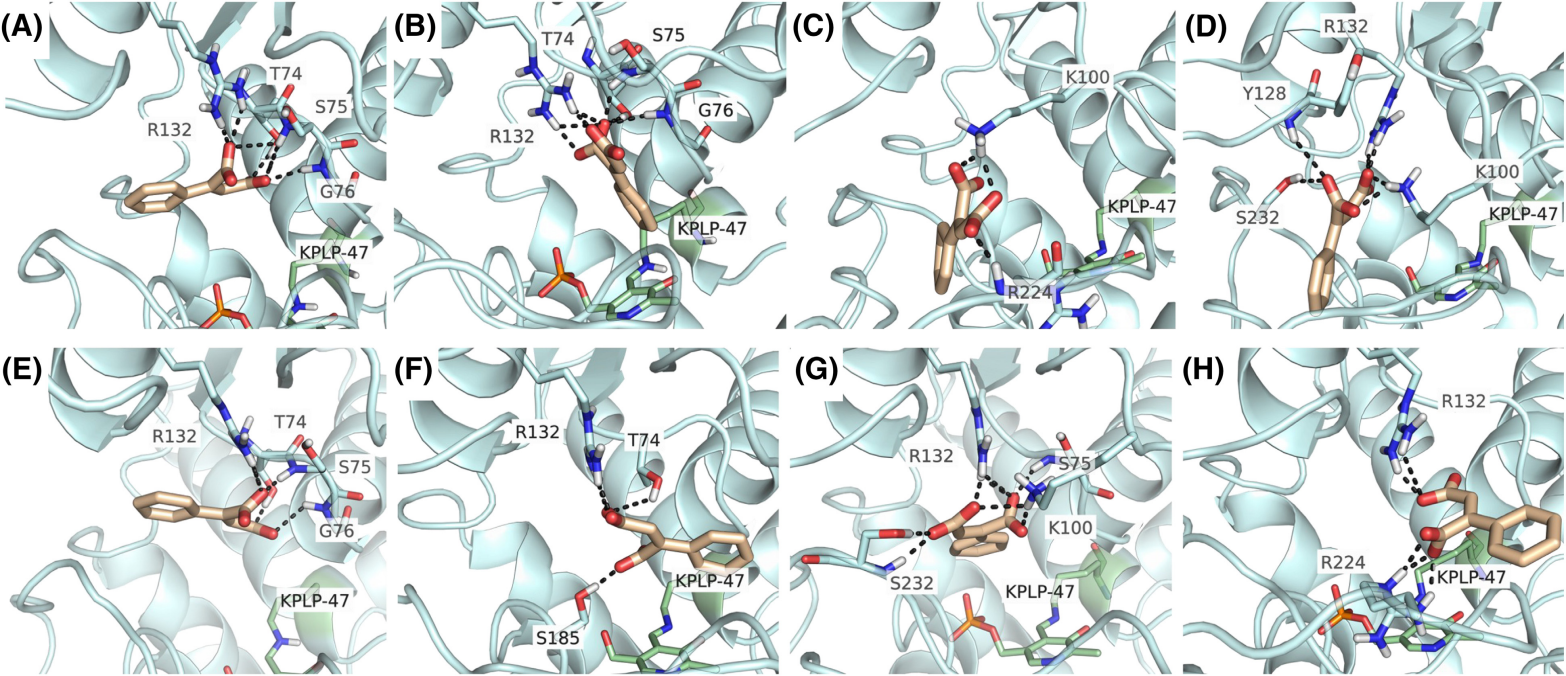
Docking pose of 2‐PhMA compared to the last frame of MD simulations. 2‐phenylmaleic acid (2‐PhMA) docking pose in (A) chain A and (E) chain B. Last frames of 2‐PhMA in chain A: (B) replica 0, (C) replica 1, (D) replica 2. Last frames of 2‐PhMA in chain B: (F) replica 0, (G) replica 1, (H) replica 2. Protein is shown in light blue cartoon, 2‐PhMA is represented in pale orange sticks, polar contacts with surrounding residues are depicted as black dashed lines.

Regarding the stability of 2‐PhMA in the active site during MD simulation, we observed different alternative binding modes (Figs. 13 and 14), which may represent prebinding, local energy minima conformations that could be relevant during the protein‐ligand interaction.

### Citrate‐derived SbnA inhibitors enter *S. aureus* cells and inhibit siderophore biosynthesis

The good inhibitory activity displayed by 2‐PhSA and 2‐PhMA encouraged the exploration of their effect on the growth of *S. aureus* and the production of siderophores. Since it is well assessed that negatively charged molecules might penetrate poorly through the bacterial cell walls, the ester derivatives of compounds **2** and **3** (**2E** and **3E**, respectively) were prepared and assayed along with the free acid forms.

#### cTMS is a suitable iron‐limiting medium to assay SbnA inhibitors

**Fig. 15:**
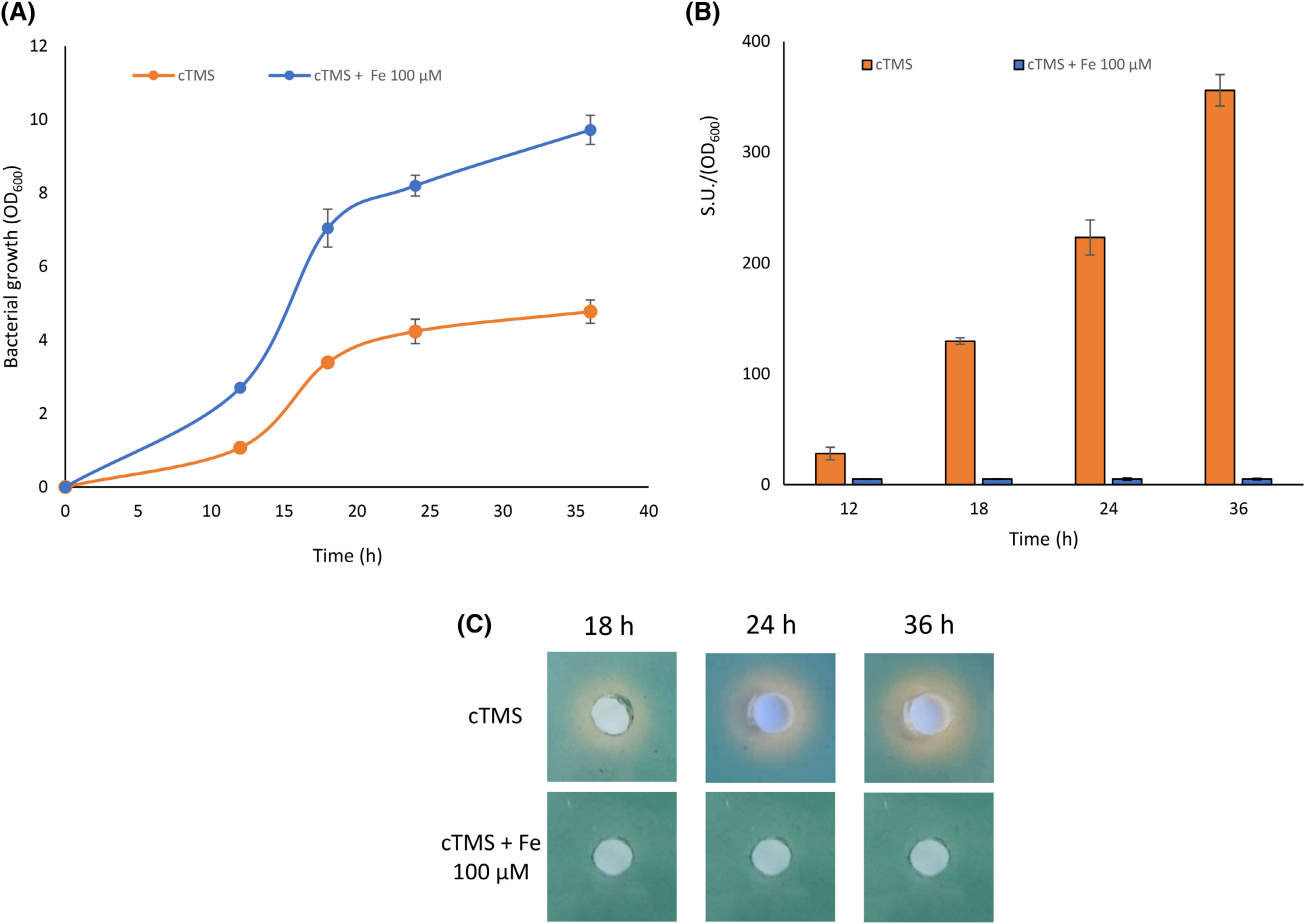
cTMS is an iron‐deprived medium suitable to investigate the activity of SbnA inhibitory compounds. (A) Growth of *S. aureus* Newman in the Chelex®‐treated Tris Minimal Succinate medium (cTMS) supplemented or not with 100 μm FeCl_3_. (B) Chrome azurol S‐Fe(III)‐hexadecyltrimethylammonium bromide (CAS) liquid and (C) solid assays were used to monitor culture supernatants for siderophore production throughout growth, at the indicated time points. All data points represent average values of three independent biological replicates, each one performed in duplicate, and error bars indicate the corresponding standard deviations from the means. S.U., Siderophore Units. CAS agar plate images are representative of three independent experiments giving similar results.

The production of SA and SB is tightly regulated by iron availability; thus, to evaluate the anti‐SbnA activity of the selected inhibitors on bacterial cells, *S*. *aureus* must be cultured under iron‐depleted conditions. To this aim, the chemically defined iron‐poor medium cTMS [25, 26] was chosen, and the growth of *S. aureus* Newman was evaluated. Bacterial cells were cultivated in cTMS and cTMS supplemented with 100 μm FeCl_3_, as the sole iron source, for up to 48 h. Growth of *S. aureus* was restricted in cTMS, since the addition of an excess of FeCl_3_ (100 μm) promoted bacterial growth, thus confirming that iron is a limiting nutrient in this medium (Fig. 15A).

Increased siderophore production is a hallmark of intracellular iron deficiency [27]; hence, the ability of *S. aureus* to produce siderophores was assessed using both the chrome azurol S‐Fe(III)‐hexadecyltrimethylammonium bromide (CAS) liquid (Fig. 15B) and solid assays (Fig. 15C). These assays consist of monitoring spectroscopically the CAS solution color change from blue to orange when a chelator removes the iron from the dye. Indeed, a ternary complex formed by CAS/Fe(III)/(HDTMA) is responsible for the blue color of the CAS solution. This complex turns orange when a stronger chelator, like siderophores, extracts Fe(III) from it. The color change is detected spectroscopically at 630 nm and by the development of an orange halo around each agar hole, containing bacterial culture supernatants, in the case of CAS liquid and solid assays, respectively.

Siderophore production in *S. aureus* began after 12‐h postinoculation and kept increasing over time in cTMS, whereas no siderophore was detected in the presence of 100 μm FeCl_3_ (Fig. 15B). Similar results were obtained by performing the CAS diffusion assay, where a large orange halo was observed around the agar well inoculated with the bacterial supernatants of *S. aureus* grown in cTMS, whereas no halo was detectable around the hole when *S. aureus* was cultured in the presence of 100 μm FeCl_3_ (Fig. 15C).

These preliminary experiments allowed us to establish cTMS as an iron‐poor medium that favors *S. aureus* siderophore production, thus being suitable for SbnA inhibitory compounds testing.

#### PhSA and PhMA methyl esters inhibit siderophore biosynthesis in *S. aureus*

**Fig. 16:**
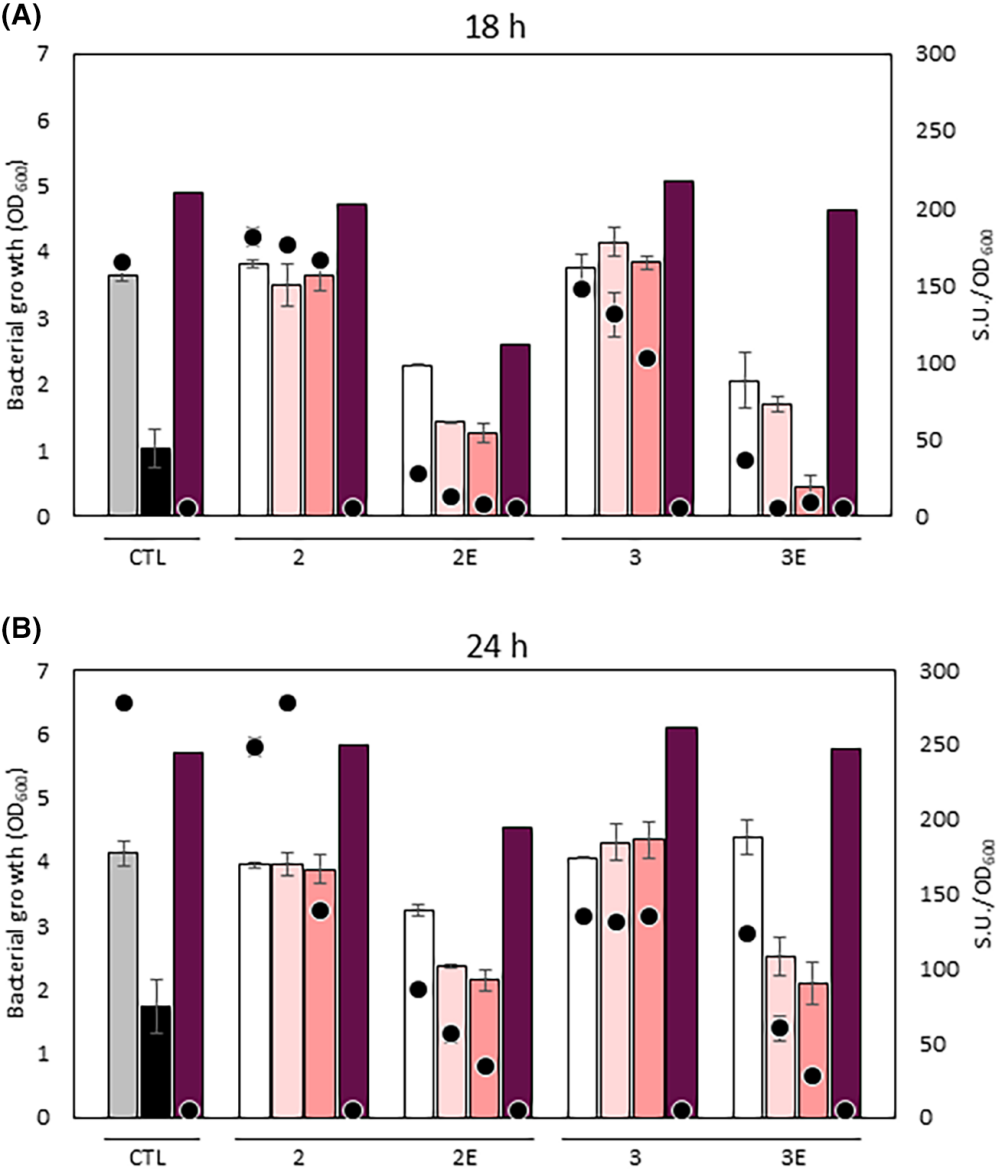
Activity of SbnA inhibitory compounds. *S. aureus* growth (bars) and siderophore production (circles) were monitored under different growth conditions at 18 h (A) and 24 h (B) post inoculum. CTL: unamended Chelex®‐treated Tris Minimal Succinate medium (cTMS), gray; cTMS supplemented with DIP, black; cTMS supplemented with 2,2′‐dipyridyl (DIP) and FeCl_3_, purple. 2‐3E: cTMS supplemented with the inhibitory molecules at different concentrations (1.5 mm white; 1.75 mm light pink; 2 mm salmon). Purple bars in the 2‐3E groups indicate cTMS supplemented with FeCl_3_ and the maximal concentration of each inhibitor (2 mm). All data points represent average values of three independent biological replicates, each one performed in duplicate, and error bars indicate the corresponding standard deviations from the means.

Since the developed inhibitors target SbnA, the first enzyme involved in SB biosynthesis, a growth deficiency and a decrease in siderophore production are expected when *S. aureus* is cultured in cTMS in the presence of sufficient amounts of the inhibitory compounds. To investigate this, the growth of *S. aureus* was monitored in cTMS supplemented with either the ester or acid form of each inhibitor at three different concentrations (i.e., 1.5–1.75 and 2 mm) and compared to that in unamended cTMS. Moreover, the growth of *S. aureus* in cTMS supplemented with the iron‐chelator 2,2′‐dipyridyl (DIP) was included as a control, to mimic the iron starvation imposed by the lack of SB (i.e., the effect of a successful SbnA inhibitor) (Fig. 16).

Interestingly, the presence of 2‐PhSA (compound **2** in Fig. 16) or 2‐PhMA (compound **3** in Fig. 16) did not affect *S. aureus* growth, at all concentrations tested both after 18‐ or 24‐h postinoculation, whereas a dose‐dependent growth reduction of *S. aureus* was observed when cultured in the presence of the corresponding esters (**2E** and **3E**, respectively) at both time points (bars in Fig. 16). To confirm that the growth defect observed was due to SbnA inhibition rather than an off‐target toxicity, growth was also monitored by adding an excess of iron to the maximal concentration of each inhibitor. Interestingly, the presence of iron not only restored the growth defect of *S. aureus* but also promoted it, in the same way as when DIP was present (Fig. 16).

The different effects of the acids and esters form of tested compounds on *S. aureus* growth could be attributed to the likely different mechanisms employed by these compounds to enter bacterial cells. Indeed, it is known that esters can cross bacterial membranes more easily, thus readily reaching their intracellular target (i.e., SbnA) and consequently affecting bacterial growth.

To further confirm the specificity of inhibitors' activity, *S. aureus* siderophore production was monitored simultaneously to the growth in cTMS and cTMS supplemented with either the acids or esters of each inhibitory compound, using the CAS liquid assay (black dots in Fig. 16). Esters dramatically reduced *S. aureus* siderophore production in a dose‐dependent manner. Surprisingly, 2‐PhMA reduced *S. aureus* siderophore production at all concentrations tested and at both time points, with a stronger effect at 24 h (*ca*. 50% reduction compared to the unamended medium), despite not affecting growth (Fig. 16B). This peculiar outcome of 2‐PhMA on *S. aureus* siderophore production may be attributed to the entrance kinetic into bacterial cells, that warrant bacterial growth at early time points (i.e., when 2‐PhMA has not yet accumulated intracellularly) but eventually affect siderophore production at later time points (i.e., when its intracellular concentration has slowly reached inhibitory concentrations).

Differently from what observed for 2‐PhMA, a minor impact on siderophore production was noticed for 2‐PhSA, and only when tested at 2 mm at 24 h (Fig. 16B). This effect might be attributed to either a different permeability of the two compounds across bacterial cells or to the different potency of the compounds on the target or to a combination of all these factors. However, this is the first report of the successful inhibition of siderophore production *in vivo* targeting SbnA and raises hope for the future development of siderophore inhibitory compounds for the treatment of infections caused by *S. aureus*.

## Conclusions

2‐PhMA identified here as an inhibitor of SbnA from *S. aureus* represents the successful discovery of an inhibitor of siderophore biosynthesis inspired by the natural modulator of the enzyme activity, citrate. Citrate has already been pointed out as a modulator of two enzymes of the SB biosynthetic pathway, SbnH and SbnC, and was identified as a ligand of SbnA [8]. Here we demonstrated that citrate binds to both the free enzyme and the α − AA intermediate, with *K*_i_s for the enzyme close to the range of its cellular concentration, thus supporting a potentially significant physiological role for the modulation of SbnA activity. The redundancy of enzymes whose activity is modulated by citrate further highlights the tight link between central metabolism and SB biosynthesis. Under iron‐repleted conditions, the Krebs cycle is fully functional and the average concentration of citrate in *S. aureus* is about 1 mm, which is sufficient to almost completely suppress the activity of SbnH and SbnC. SbnA is also inhibited under these conditions, albeit not completely. This suggests that SbnA is the most responsive to citrate fluctuations, since both an increase and a decrease of its concentration will lead to a proportional increase or decrease of SbnA activity. The removal of one carboxylate from citrate and the addition of a phenyl substituent at the 2‐position (2‐PhSA) leads to a sevenfold increase in potency, and the further addition of a double bond (2‐PhMA) to an additional improvement that, overall, leads to a 50‐fold increase in inhibition potency with respect to citrate. The binding pose of 2‐PhMA shows interactions with the l‐OPS binding site that are analogous to those established by the α‐AA in PDB ID: 5D85. As suggested by MD simulations, further modulations may be needed to stabilize the binding pose of the 2‐PhMA fragments in the SbnA site, suggesting that further chemical modifications will be possible to improve the inhibitor properties. Furthermore, a conformation representing an intermediate state between the SbnA open and closed states was stabilized during MD simulations, regardless of the presence of 2‐PhMA, that might be relevant for inhibitor design. The specificity of the designed SbnA inhibitors deserves further investigation in the future. Indeed, the inhibitors similarity to citrate, which sets the basis for their mechanism of inhibition, might potentially be responsible for off‐target toxicity, since citrate‐like molecules are the substrates of many enzymes, both human and microbial. *In vitro* toxicity assays will be essential to validate the safety profile of these inhibitors and guide the next stages of their development. This approach will help ensure that promising lead compounds can be optimized while minimizing unwanted interference with host cellular processes. 2‐PhMA and 2‐PhSA penetrate poorly through the bacterial cell wall, but their diffusion is not fully hampered, as demonstrated by a small, but reproducible, inhibitory effect on siderophore production. Interestingly, the ester counterparts of the two inhibitors are likely to penetrate better and, indeed, the effect on siderophore production is stronger and concentration‐dependent. Under the conditions tested here it is not possible to detect a significant difference in the potency of the two compounds, likely because the poor permeation through the cell wall levels off any effect of compound potency on the production of siderophores. Further developments for inhibitors of SbnA may involve modulation of 2‐PhMA with a ligand‐based approach, exploiting its rigidity and exploring different substitutions on the phenyl moiety to increase size and specificity. Notably, heme is abundant in damaged tissues during infection and can serve as an alternative iron source, allowing *S. aureus* to persist at the infection site even when siderophore production is disrupted [28]. Therefore, the ability of *S. aureus* to acquire iron through heme‐acquisition systems such as the Isd (iron‐regulated surface determinant) pathway [29, 30] must be carefully investigated in the presence of siderophore inhibitors in preclinical models of infections.

## Materials and methods

### SbnA recombinant production and characterization

#### Reagents

Hepes and sodium chloride were purchased from PanReac Applichem ITW Reagents (Darmstadt, Germany). Tris base was purchased from VWR International Srl (Milano, MI, Italy) and 7methyl‐6‐thioguanosine (MESG) was purchased from Cayman (Ann Arbor, MI, USA). (S)‐(+)‐2,3‐diaminopropionic acid hydrochloride (l‐Dap) was purchased from TCI Europe N.V. (Zwijndrecht, Belgium), Tris(2‐carboxyethyl) phosphine hydrochloride (TCEP) from Apollo Scientific (Stockport, UK) and citrate from Carlo Erba Reagents (Cornaredo, MI, Italy). The malachite green reagent was purchased from ChemCruz® (Santa Cruz Biotechnology Inc., Dallas, TX, USA), while purine nucleoside phosphorylase (PNP) and all the remaining chemicals were purchased from Merck (Darmstadt, Germany). All reagents were used as received, unless otherwise specified.

#### Protein expression and purification

The synthetic gene of *sbnA* from *S. aureus* (UniProt entry A6QDA0) optimized for *Escherichia coli* expression was purchased from Thermo Fisher Scientific (Invitrogen GeneArt Gene Synthesis services). A sequence encoding for the TEV protease cleavage site was added at the N terminus. The sequence was inserted in the BamHI/XhoI sites of the pSH21p::TrxA vector [31], which allows to clone the sequence *in frame* to the sequence coding for HisTag‐Thioredoxin at the N terminus.

SbnA was over‐expressed in *E. coli* BL21 ArcticExpress (DE3) cells (Agilent Technologies, Santa Clara, CA, USA) grown at 30 °C for 3 h in 0.5 L of LB medium supplemented with 100 μg·mL^−1^ ampicillin and 20 μg·mL^−1^ gentamycin. The temperature was then reduced to 4 °C and, after 20 min, expression was induced by adding 1 mm isopropyl‐β‐d‐1‐thiogalactopyranoside (IPTG) at 4 °C for 24 h. Cells were collected by centrifugation (10 000 ***g*** for 10 min at 4 °C) and stored at −20 °C.

The thawed pellet was resuspended and incubated in lysis buffer (50 mm Tris, 100 mm NaCl, 1 mm TCEP, 0.2 mm PLP, 1 mg·mL^−1^ lysozyme, 0.2 mm PMSF, 0.2 mm benzamidine and 1.5 μm pepstatin, pH 8.0 at 4 °C) for 1 h, sonicated, and centrifuged (18 000 ***g*** for 1 h at 4 °C) to separate soluble from insoluble fractions. The purification of SbnA was performed by Immobilized Metal Affinity Chromatography (IMAC) on a Talon Superflow™ resin (Cytiva, Marlborough, MA, USA). Two wash steps were performed at 10 and 20 mm imidazole in Tris buffer (50 mm Tris, 100 mm NaCl, 1 mm TCEP, pH 8.0 at 4 °C), while SbnA elution was performed in the Tris buffer containing 250 mm imidazole. The removal of the N‐terminal tag was performed on the purified protein solution adding a fivefold molar excess PLP with TEV protease in a 1 : 100 weight ratio with respect to SbnA. The solution was dialyzed overnight at 4 °C against the Tris buffer to remove imidazole. The solution was then loaded on a Talon Superflow™ resin to purify untagged SbnA, which was then concentrated up to 1.8 mg·mL^−1^, aliquoted, and stored at −80 °C. The protein yield was 2.8 mg per liter of culture.

#### Spectroscopic characterization

##### UV–visible spectroscopy

Absorption spectra were collected in a quartz microcuvette of 1 cm path length using a Cary4000 spectrophotometer (Agilent Technologies). Measurements were carried out in 50 mm HEPES buffer, pH 7.8 at 25 °C. SbnA concentration (in monomers) was estimated using an extinction coefficient at 280 nm of 34 380 m ^−1^·cm^−1^, calculated by the protparam server (web.expasy.org/protparam/).

##### Fluorescence spectroscopy

Fluorescence emission spectra of SbnA tryptophan residues upon excitation at 298 nm were collected in a 3 mm path length quartz microcuvette on a Fluoromax‐3 (Horiba Jobin Yvon, Kyoto, Japan) spectrofluorometer equipped with a thermostated water bath. Measurements were carried out in a 50 mm HEPES buffer, pH 7.8 at 20 °C.

#### Activity assays

The activity of SbnA was measured by the PNP‐coupled continuous assay [32] at 25 ± 0.4 °C in a reaction buffer containing 50 mm HEPES pH 7.8, 1 mm TCEP. The assay exploits the phosphate produced by the activity of SbnA that is used by PNP to catalyze the phosphorolysis of MESG to 2‐amino‐6‐mercapto‐7‐methylpurine (AMMP) and ribose 1‐phosphate. The production of AMMP is monitored at 360 nm. The preparation of PNP and MESG stock solutions and the evaluation of the PNP‐specific activity were carried out as described in [32]. The concentration of MESG was calculated from its absorbance at 331 nm using an extinction coefficient of 32 000 m ^−1^·cm^−1^. The continuous assays were performed in reaction buffer with the addition of 100 μm MESG. The dependence of the reaction rate on the coupled enzyme concentration was measured at 50 nm SbnA, 0.72 mm l‐OPS, and 30 mm l‐Glu (i.e., almost at saturating concentrations of both substrates) and varying the amount of PNP between 216 and 1080 mU. The relationship between the reaction rate and PNP concentration was linear within the tested range; thus, 216 mU PNP was used in the following assays. The dependence of the reaction rate on SbnA concentration was evaluated in the same condition reported above (0.72 mm l‐OPS and 30 mm l‐Glu) within the 25–100 nm range. For the calculation of the catalytic parameters, different concentrations of substrates were used ranging from 0.035 to 0.720 mm for l‐OPS and from 0.1 to 150 mm for l‐Glu. Each reaction was performed in a quartz microcuvette of 1 cm path length and preincubated for 2 min at 25 °C in the cuvette holder of a Cary4000 spectrophotometer, before reading the baseline absorbance for 1–2 min at 360 nm. The reactions were then triggered by the addition of SbnA. The initial velocity and the rate of ribose 1‐phosphate and 2‐amino‐6‐mercapto‐7‐methylpurine (AMMP) production were calculated as described in [32]. For the evaluation of SbnA kinetic mechanism, the activity was measured at concentrations of l‐Glu ranging from 0.1 to 150 mm, keeping the concentration of l‐OPS fixed at 0.72, 0.08, or 0.035 mm. Michaelis Menten plots were then globally fitted using a double displacement equation with inhibition by one substrate as reported in [33] (Eqn 1), where *v* is the initial velocity, *V*_max_ is the initial velocity at substrate saturation, [l‐OPS] and [l‐Glu] are the concentrations of l‐OPS and l‐Glu, *K*_M l‐OPS_ and *K*_M l‐Glu_ refer to the Michaelis Menten constants of l‐OPS and l‐Glu, respectively, and *K*_i l‐Glu_ represents the inhibition constant for l‐Glu.

(1) $$v_{0}=\frac{V_{\max}\left[L‐\mathrm{OPS}\right]\left[L‐\mathrm{Glu}\right]}{K_{M\;L‐\mathrm{OPS}}\left[L‐\mathrm{Glu}\right]\left(1+\frac{\left[L‐\mathrm{Glu}\right]}{K_{i\;L‐\mathrm{Glu}}}\right)+K_{M\;L‐\mathrm{Glu}}\left[L‐\mathrm{OPS}\right]+\left[L‐\mathrm{OPS}\right]\left[L‐\mathrm{Glu}\right]}.$$

### Molecular modeling

Molecular modeling and simulation methods including Induced Fit Docking (IFD) and molecular dynamics simulations (MD) have been performed to acquire details on the inhibitory activity of selected molecules against SbnA at the molecular scale and to rationalize the potency ranking observed.

#### Structural models

Docking and molecular dynamics were carried out starting from SbnA (code 5D84) with PLP bound as an internal aldimine to the catalytic Lys47 (Fig. 3). In this structure, the active site is open but the orientation of PLP provides an energetically favorable site for potential inhibitors to be docked.

#### Docking

Docking was performed using the Maestro Schrödinger software version 2021–2. Citrate, 2‐phenylsuccinic acid (2‐PhSA) and 2‐phenylmaleic acid (Z‐2‐phenylbut‐2‐enedioic acid, 2‐PhMA) were prepared at pH 8.0 with the Ligprep module, and docked in the binding site of both monomers of the open structure 5D84 by means of IFD [34, 35, 36]. The grid boxes for the binding sites of each monomer were built considering the PLP as the centroid. During the initial docking procedure, the van der Waals scaling factor was set at 0.5 for both the receptor and ligand. The Prime refinement step was set on side chains of residues within 5 Å of the ligand.

#### MD simulations

Amber99sb‐ILDN force field for the protein groups and ions [37] and TIP3P water model [38] were adopted for parameterization. The parameters for *N*′‐pyridoxal‐lysine‐5′‐monophosphate (KPLP), the internal aldimine of the cofactor with the active site lysine were added to the Amber‐ILDN library based on previous methods [39, 40]. The partial charges were taken from the RESP ESP charge Derive Server Development (pyR.E.D) server [41] which uses the RESP method. A number of missing angles, dihedrals, and improper dihedrals were modified or added according to Acpype parametrization [42, 43] (Table S1) to mimic the double bond behavior more accurately. The charges and bonded interactions for 2‐PhMA were calculated using biki life sciences software by restrained electrostatic potential density functional theory method (RESP‐DFT) [44].

Long‐range interactions were evaluated using particle‐mesh Ewald (PME) summations with a cutoff of 12 Å for the real part. The Lennard‐Jones interactions were truncated at 12 Å with an atom‐based force‐switching function, effective at 10 Å. Hydrogen bonds were constrained by the LINear Constraint Solver (LINCS) algorithm [45]. The integration time step was set to 2 fs.

Water molecules were added to the protein–ligand complex in a cubic simulation box of about 10.28 nm^3^. The system was neutralized by adding Na^+^ ions. Following one step of steepest descent minimization by setting the maximum energy tolerance to 100.0 kJ·mol^−1^, the system underwent thermalization for six steps at temperatures ranging from 50 to 300 K. The equilibration was run for 1 ns in the NVT ensemble at 300 K, and then for another 1 ns in the NPT ensemble, adapting the pressure to 1 atm using v‐rescale thermostat [46] and Berendsen barostat [47]. During thermalization and pressure adaptation, the protein heavy atoms were kept fixed by positional restraints with a force of 1000 kJ·mol^−1^·nm^−1^which were then gradually released. After the equilibration phase, 1 μs long simulations were performed on 3 identical replicas using the v‐rescale thermostat [46] with an effective relaxation time of 0.1 ps, an isotropic Parrinello‐Rahman barostat [48], and a pressure coupling effective every 2 ps with a compressibility of 4.5 × 10^−5^·bar^−1^ to confirm the stability of 2‐PhMA in the pocket. MD simulations and analyses were carried out in gromacs v. 2023.3 Royal Institute of Technology and Uppsala University, Sweden)(, figures were produced in pymol v. 2.6.1 (Schrödinger LLC comapny, NewYork, USA).

### Identification and activity of inhibitors

Aqueous stock solutions of citrate, α‐ketoglutarate (α‐KG) and l‐serine were prepared at 100 mm, while the l‐Dap stock solution was solubilized at 360 mm. When needed, the pH was adjusted to neutrality. Tricarballylic acid (CAS 99‐14‐9) and 2‐PhSA (CAS 635‐51‐8) were purchased from Specs (Zoetermeer, The Netherlands) and used for initial inhibition assays. 2‐PhSA purchased from Merck (Darmstadt, Germany) was used for the determination of *K*_i_ and the inhibition mechanism. 2‐PhMA (CAS 16110‐98‐8) was purchased from Chemspace® (Riga, Latvia). The compounds were solubilized in water at either 50 or 100 mm; gentle heating and sonication were applied when needed.

#### Compound screening

The activity assays for compound screening were carried out at 25 ± 0.4 °C in a reaction buffer containing 100 mm HEPES pH 7.8, 1 mm TCEP by a discontinuous activity assay. The solutions in the absence and the presence of selected compounds were preincubated at 25 ± 0.4 °C in the reaction buffer (see above) in the presence of substrates concentration equal to the *K*_m_ (i.e., 0.03 mm l‐OPS and 3 mm l‐Glu). The reactions were triggered by the addition of SbnA at a final concentration of 70 nm. 20 μL aliquots were taken at time 0 and after 10 min and dispensed in a 96‐well microplate (Sarstedt AG & Co KG, Nümbrecht, Germany) containing 2 μL of 10% (m/v) trichloroacetic acid (TCA) to stop the reaction. 100 μL of malachite green reagent was added to the samples and the mixtures were incubated for 30 min at room temperature in the dark. The absorbance of the complex between the malachite green reagent and inorganic phosphate was measured at 620 nm using a HALO LED 96 microplate reader (Dynamica, Livingstone, UK). The amount of phosphate produced by the enzyme activity was calculated using a calibration curve from 0 to 100 μm phosphate. The relative activity of the enzyme in the presence of inhibitors was calculated as a fraction of phosphate produced with respect to the uninhibited reaction. All measurements were performed in duplicate.

#### Determination of the potency and the mechanism of action of inhibitors

The IC_50_ values of inhibitors were calculated by measuring the SbnA activity exploiting the PNP‐coupled assay (2.1.4.1) at l‐OPS and l‐Glu concentrations equal to the *K*_m_ (i.e., 0.03 mm l‐OPS and 3 mm l‐Glu) and different compounds' concentrations, ranging from 0.25 to 15 mm for α‐KG, 0.1 to 10 mm for citrate, 0.1 to 5 mm for 2‐PhSA, and from 0.005 to 1 mm for the 2‐PhMA. The data were then fitted to a binding isotherm (Eqn 2), where *v*_*i*_ is the initial velocity in the presence of different inhibitors concentrations, *v*_*0*_ is the initial velocity in the absence of inhibitors, and IC_50_ is the inhibitor concentration corresponding to half of the *v*_*0*_.

(2) $$\frac{v_{i}}{v_{o}}=\frac{{\mathrm{IC}}_{50}}{\left[I\right]+{\mathrm{IC}}_{50}}$$

The inhibition mechanisms of citrate, 2‐PhSA, and 2‐PhMA were evaluated by titration of one of the two substrates at a fixed nonsaturating concentration of the other substrate in the presence of different, fixed concentrations of inhibitors. Michaelis–Menten plots at different citrate concentrations were globally fitted to the equation for a ping‐pong enzymatic reaction with added inhibitor that binds to E and F enzyme forms, either varying substrate A (Eqn 3) or substrate B (Eqn 4) [33].

(3) $$v_{0}=\frac{V_{\max}\left[L‐\mathrm{OPS}\right]}{K_{m\;L‐\mathrm{OPS}}\left(1+\frac{\text{[citrate]}}{K_{i1}}\right)+\left[L‐\mathrm{OPS}\right]\left(1+\frac{K_{m\;L‐\mathrm{Glu}}}{\left[L‐\mathrm{Glu}\right]}\right)\left[1+\frac{\text{[citrate]}}{K_{i2}\left(1+\frac{\left[L‐\mathrm{Glu}\right]}{K_{m\;L‐\mathrm{Glu}}}\right)}\right]}.$$

(4) $$v_{0}=\frac{V_{\max}\left[L‐\mathrm{Glu}\right]}{K_{m\;L‐\mathrm{Glu}}\left(1+\frac{\text{[citrate]}}{K_{i2}}\right)+\left[L‐\mathrm{Glu}\right]\left(1+\frac{K_{m\;L‐\mathrm{OPS}}}{\left[L‐\mathrm{OPS}\right]}\right)\left[1+\frac{\text{[citrate]}}{K_{i1}\left(1+\frac{\left[L‐\mathrm{OPS}\right]}{K_{m\;L‐\mathrm{OPS}}}\right)}\right]}.$$

where *v*_*0*_ is the initial velocity, *V*_max_ is the initial velocity at substrate saturation, [l‐OPS], [l‐Glu] and [citrate] are the concentrations of l‐OPS, l‐Glu, and citrate, respectively, *K*_M l‐OPS_ and *K*_M l‐Glu_ refer to the Michaelis–Menten constants of l‐OPS and l‐Glu, respectively, while *K*_i1_ and *K*_i2_ represent the inhibition constants of citrate for the free enzyme and the α‐aminoacrylate intermediate, respectively.

To improve the fitting, the calculated *K*_m_ values of both substrates (*K*_m_ = 0.027 mm for l‐OPS and *K*_m_ = 3.25 mm for l‐Glu) were added as constrained values in the equation.

For 2‐PhSA and 2‐PhMA, Michaelis–Menten plots at different concentrations of the inhibitor were globally fitted to the equation for competitive inhibition for the free enzyme of a ping‐pong enzymatic reaction varying substrate A (Eqn 5) [33].

(5) $$v_{0}=\frac{V_{\max}\left[L‐\mathrm{OPS}\right]}{K_{m\;L‐\mathrm{OPS}}\;\left(1+\frac{\left[I\right]}{K_{i}}\right)+\left[L‐\mathrm{OPS}\right]\left(1+\frac{K_{m\;L‐\mathrm{Glu}}}{L‐\mathrm{Glu}}\right)}.$$

where *v*_*0*_ is the initial velocity, *V*_max_ is the initial velocity at substrate saturation, [l‐OPS], [l‐Glu], and [*I*] are the concentrations of l‐OPS, l‐Glu, and inhibitors, respectively, *K*_M l‐OPS_ and *K*_M l‐Glu_ refer to the Michaelis–Menten constants of l‐OPS and l‐Glu, respectively, while *K*_i_ represents the inhibition constant of the inhibitors for the free enzyme.

To improve the fitting, the calculated *K*_m_ values of both substrates were added as constrained values in the equation.

#### Esterification of selected compounds

Compounds used for microbiological assays were esterified to improve permeation in *S. aureus* cells.

##### Dimethyl 2‐phenylsuccinate, 2E

**Fig. 17:**
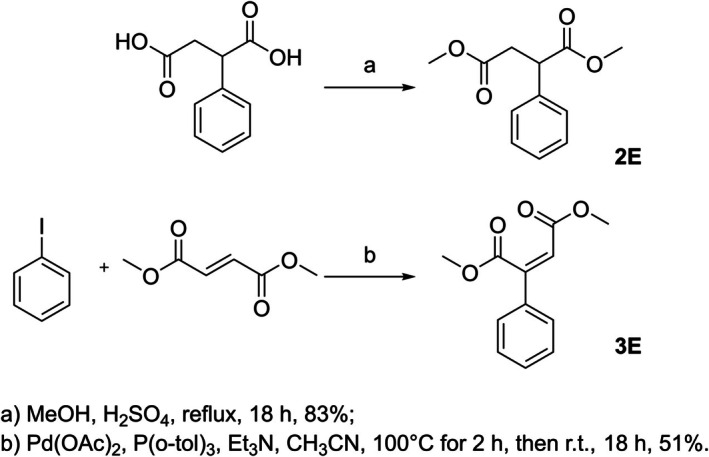
Synthesis of esters used for microbiological assays. Reaction conditions for (a) dimethyl 2‐phenylsuccinate (2E) and (b) dimethyl 2‐phenylmaleate (3E).

The methyl ester **2E** was obtained by esterification of commercial phenylsuccinic acid in an acidic methanol solution at reflux overnight (Fig. 17).

20 mL of a methanolic solution of 2‐PhSA (2.00 g, 10.3 mmol) was added with 5 drops of concentrated H_2_SO_4_, and the reaction mixture was refluxed for 18 h. After completion, the solvent was removed under reduced pressure. The residue was diluted with CH_2_Cl_2_ (20 mL) and washed with saturated NaHCO_3_ solution (3 × 25 mL), dried over Na_2_SO_4_, and concentrated to dryness. The desired compound was obtained as a transparent oil without further purification (1.9 g, 83%).

^1^H NMR (600 MHz, CDCl_3_) δ 7.35–7.31 (m, 2H), 7.29–7.26 (m, 3H), 4.09 (dd, J = 10.2, 5.2 Hz, 1H), 3.68 (s, 6H), 3.21 (dd, J = 17.0, 10.2 Hz, 1H), 2.67 (dd, J = 17.0, 5.2 Hz, 1H).

^13^C NMR (150 MHz, CDCl_3_) δ 173.4, 1712.0, 137.6, 128.9, 127.7, 127.7, 52.4, 51.9, 47.0, 37.6.

ESI‐MS [M + H]^+^: m/z 223.2.

##### Dimethyl 2‐phenylmaleate, 3E

The methyl ester **3E** was obtained starting from iodobenzene by arylation of dimethyl fumarate with tri(o‐tolyl)phosphine–palladium acetate catalysts, stereospecifically via a *syn* addition of the organopalladium intermediate followed by a *syn* ‘palladium hydride’ elimination [49] (Fig. 17).

A mixture of iodobenzene (2 g, 9.8 mmol), dimethyl fumarate (1.8 g, 12.25 mmol), Pd(OAc)_2_ (0.022 g, 0.098 mmol), P(o‐tol)_3_ (0.060 g, 0.196 mmol), and Et_3_N (0.991 g, 9.8 mmol) was stirred under N_2_ at 100 °C in CH_3_CN (7 mL) for 2 h. After this time, the reaction was stirred at r.t. overnight. Upon completion, the solvent was evaporated *in vacuo*, the residue was diluted with CH_2_Cl_2_ (20 mL) and washed with H_2_O (3 × 25 mL), dried over Na_2_SO_4_, and concentrated to dryness. Purification of the residue by flash chromatography, using PE/EtOAc (95/5 v/v) as the eluent, gave the target compound as an orange oil (1.1 g, 51%).

^1^H NMR (600 MHz, CDCl_3_) δ 7.50–7.46 (m, 2H), 7.45–7.38 (m, 3H), 6.32 (s, 1H), 3.95 (s, 3H), 3.79 (s, 3H).

^13^C NMR (150 MHz, CDCl_3_) δ 168.3, 165.4, 149.0, 133.1, 130.6, 128.9, 126.8, 116.9, 52.7, 52.0.

ESI‐MS [M + H]^+^: m/z 221.2.

### Bacterial culture conditions and siderophore detection

*S. aureus* Newman [50] was routinely cultured in Tryptic Soy Agar (TSA) at 37 °C for 18 h and stored with 15% glycerol (v/v) at −80 °C. In this study, a chemically defined minimal medium, namely Tris Minimal Succinate (TMS), was prepared according to a previously described protocol [25], with minor modifications. Briefly, all glassware used to prepare the medium was pretreated with 0.1 m HCl for 24 h and washed at least three times with Double Distilled H_2_O (ddH_2_O) to avoid iron contamination. Once the medium was prepared, TMS was then treated with 5% (w/v) Chelex® resin (Bio‐Rad, Hercules, CA, USA) at 4 °C to chelate all trace metals. After Chelex® removal by filtration (Whatman no. 1 paper filter, Merck), 1 mL of MgCl_2_ (95.3 mg·mL^−1^), and 1 mL of CaCl_2_ (11.1 mg·mL^−1^) were incorporated, and the pH of the medium was adjusted to 7.4, then sterilized by filtration using the Corning® filter system (Corning Inc., New York, NY, USA) and stored at 4 °C, yielding cTMS.

When required, DIP was prepared as a 30 mm stock solution in ddH_2_O and stored at −20 °C. FeCl_3_ was prepared as a 0.1 m stock solution in 10 mm HCl and stored at −20 °C. The acidic form of either 2‐PhMA or 2‐PhSA was freshly prepared in cTMS at 100 mm and sterilized by filtration, while their corresponding esters, namely compounds **3E** and **2E**, were prepared in dimethyl sulfoxide (DMSO) as a stock solution at 50 and 100 mm, respectively. DIP, FeCl_3_, and inhibitor molecules were added to cTMS at the required final concentrations, as indicated.

The ability of *S. aureus* to produce iron chelators (siderophores) was investigated using the chrome azurol S‐Fe(III)‐hexadecyltrimethylammonium bromide (CAS) method both in liquid and solid media [51]. Briefly, bacterial cells were grown in cTMS and cTMS supplemented with either FeCl_3_ or inhibitor compounds, which were added at different concentrations. Cultures were then centrifuged at 5000 ***g*** for 10 min at 4 °C.

For the liquid assay, 100 μL of the bacterial culture supernatants were serially diluted in a 96‐well microtiter plate. The assay was then performed by adding 100 μL of CAS reagent to the culture supernatant and 2 μL of CAS shuttle solution (50 mg·mL^−1^ of 5‐sulfosalicylic acid). cTMS was used as a reference. Absorbance at 630 nm was measured in a multiplate reader (SPARK 10M TECAN®, Männedorf, Switzerland) after 45 min of incubation in the dark at room temperature. Siderophore units (S.U.) were determined from absorbance at 630 nm according to Eqn (6):

(6) $$S.U.=\left[\frac{\left(A_{r}-A_{s}\right)}{A_{r}}\right]\cdot 100$$

where *A*_*r*_ is the reference absorbance of cTMS, while *A*_*s*_ is the sample absorbance. S.U. were normalized to the initial culture turbidity (measured at OD_600_), considering the dilution factor, as follows: (S.U./OD_600_) × dilution factor.

For the CAS agar diffusion assay [52], CAS plates were punched with 5‐mm‐diameter holes by using a gel puncher, and 10 μL of bacterial supernatant was inoculated in each well. Plates were then incubated for up to 48 h at 37 °C. The halo around each agar hole provided a qualitative estimation of the amount of released siderophores.

### Activity testing of the SbnA inhibitory molecules

Since the *sbnA* gene expression is influenced by the presence of oxygen in the culture medium, as the gene is more expressed under good aeration [53], the activity of the inhibitory molecules on *S. aureus* wild‐type strain was assessed in macro‐volumes (7 mL of cTMS in a 50 mL tube). For that, *S. aureus* was cultured O/N at 37 °C in cTMS supplemented with 300 μm DIP with vigorous shaking (200 r.p.m.). Bacterial cells were washed once and then normalized to OD_600_ = 0.001 in 7 mL of cTMS ±100 μm DIP supplemented or not with 100 μm FeCl_3_. Where indicated, the inhibitory compounds were added at 1.5, 1.75, or 2 mm. Cultures were incubated at 37 °C with vigorous shaking, and growth was measured periodically by turbidimetry (OD_600_) for up to 36 h.

## Conflict of interest

The authors declare no conflict of interest.

## Author contributions

EF, FS, BC, and SB conceived the study; SH, ODB, FM, MM, EG, and LL conceived the experiments and analyzed data; SH, MC, SA, FM, MF, and GT performed the experiments and analyzed data; SF, LR, MM, LL, and BC supervised the study; FS, EF, and SB acquired funds; BC and EF drafted the original manuscript; SH, MC, ODB, FM, and EG curated the visualization; SF, FM, MM, and SB revised the original draft; all the authors read and corrected the manuscript.

## Supporting information


**Table S1.** Modified Force field parameters for the C4A‐NZ double bond.

**Movie S1.** MD simulation of SbnA in its free form.

**Movie S2.** MD simulation of SbnA bound to 2‐PhMA inhibitor.

## Data Availability

The data that support the findings of this study are available from the corresponding author [barbara.campanini@unipr.it] upon reasonable request.
