# Supplementary material for: First‐in‐class inhibitors of SbnA reduce siderophore production in Staphylococcus aureus
Source: FEBS J. 2025 Apr 2;292(17):4650–74. doi: 10.1111/febs.70076 (PMC12414870; doi:10.1111/febs.70076)
Supplement: Supplementary file 1 — Table S1. Modified Force field parameters for the C4A‐NZ double bond. [file FEBS-292-4650-s003.pdf]

SUPPORTING INFORMATION

Table S1. Modified Force field parameters for the C4A-NZ double bond.

**Bond stretch**			
Bond	**r_{ij}(nm)**	**K_r(kJ/mol.nm^2)**	
NZ-C4A	0.13350	410032.0	
**Angle bend**			
Angles	**θ_{ijk}(degree)**	**K_θ(kJ/mol)**	
C4A-NZ-CE	**123.00**	**635.6300**	
NZ-C4A-H4	**115.06**	**406.270**	
**Torsion**			
Dihedrals	**ϕ_{ijkl}(degree)**	**K_ϕ(kJ/mol)**	**n**
NZ-C4A-C4-C3, NZ-C4A-C4-C5	**180.00**	**2.82360**	**2**
HZ-NZ-C4A-H4	**180.00**	**4.39320**	**2**
HZ-NZ-CE-HE2	**0.000**	**2.80328**	**1**
CA-C4A-NZ-HZ	**180.00**	**4.39320**	**2**
H4-C4A-C4-C3	**180.00**	**2.92880**	**2**
H4-C4A-C4-C3	**180.00**	**2.92880**	**2**
H4-C4A-NZ-CE	**180.00**	**4.39320**	**2**
CE-NZ-C4A-C4	**180.00**	**4.97896**	**2**
Improper dihedrals	**ϕ_{ijkl}(degree)**	**K_ϕ(kJ/mol)**	**n**
C2-C4-C3-O3	**180.00**	**4.393200**	**4**
C3-C5-C4-C4A	**180.00**	**0.460240**	**4**
CT-C4A-NZ-HZ	**180.00**	**0.460240**	**4**

Movie 1. MD simulation of SbnA in its free form. MD replica (1 microsecond) of 2-PhMA (light yellow sticks) in SbnA (light blue). The starting docking pose of 2-PhMA is shown as violet sticks for comparison, PLP in the internal aldimine state is depicted in light green sticks. This trajectory corresponds to the replica 'rep_0' in Figures 11-14.

Movie 2. MD simulation of SbnA bound to 2-PhMA inhibitor. MD replica (1 microsecond) of the free form of the SbnA enzyme (light blue). The X-ray structure of SbnA in complex with the α-aminoacrylate intermediate (PDB ID: 5d85, teal) is superposed for comparison. This trajectory corresponds to the replica 'rep_0' in Figures 11-14.
